# Induction of apoptosis and hypoxic stress in malignant melanoma cells via graphene-mediated far-infrared radiation

**DOI:** 10.1186/s12885-025-14031-0

**Published:** 2025-04-07

**Authors:** Wumei Zhao, Ziwen Chen, Wenxing Fu, Chenyan Ye, Haijing Fu, Tianyi Xu, Binghui Wu, Lina Chen, Shi-Jun Shan

**Affiliations:** 1 Department of Dermatology, School of Medicine, Xiang’an Hospital of Xiamen University, Xiamen University, Xiamen, 361101 China; 2 New Cornerstone Science Laboratory, State Key Laboratory for Physical Chemistry of Solid Surfaces, Collaborative Innovation Center of Chemistry for Energy Materials, and National & Local Joint Engineering Research Center of Preparation Technology of Nanomaterials, College of Chemistry and Chemical Engineering, Xiamen University, Xiamen, 361005 China; 3 Innovation Laboratory for Sciences and Technologies of Energy Materials of Fujian Province (IKKEM), Xiamen, 361101 China; 4 Department of cardiology, Shaoxing Central Hospital, Shaoxing, 312030 China; 5 Jinhua Fifth Hospital, College of Mathematical Medicine, Zhejiang Normal University, Jinhua, 321004 China

**Keywords:** Malignant melanoma, Far-infrared radiation, Cell apoptosis, Cell cycle, Hypoxic stress

## Abstract

**Background:**

Malignant melanoma (MM) is a highly aggressive skin tumor with a rising incidence and poor prognosis. Although current clinical treatments, including surgery, targeted therapy, immunotherapy, and radiotherapy, have shown some efficacy, therapeutic options remain limited for elderly patients and those with metastatic disease, highlighting the urgent need for novel therapeutic strategies. In recent years, the unique far-infrared radiation (FIR) properties of graphene have demonstrated potential applications in cancer treatment. However, the mechanisms underlying FIR’s effects in MM therapy remain poorly understood.

**Methods:**

This study systematically evaluated the inhibitory effects of FIR on MM through in vitro cell experiments, animal models, and molecular mechanism analysis. First, the B16F10 melanoma cell line was used as the experimental model. The effects of FIR on cell proliferation, apoptosis, and the cell cycle were assessed using CCK-8 assays and flow cytometry, while RNA sequencing was conducted to analyze the associated signaling pathways. Second, specific caspase inhibitors were employed to further validate the mechanisms of FIR-induced apoptosis. Finally, a syngeneic tumor transplantation model in C57BL/6J mice was established to comfirm the anti-tumor efficacy of FIR in vivo, thereby comprehensively elucidating its anti-cancer mechanisms.

**Results:**

The results demonstrated that FIR significantly inhibits MM. In vitro experiments revealed that FIR treatment markedly suppressed B16F10 cell proliferation, induced apoptosis, caused G0/G1 phase cell cycle arrest, and downregulated the expression of hypoxia-related proteins such as HIF-1α. In animal studies, FIR significantly inhibited tumor growth. RNA sequencing revealed that FIR exerts its anti-cancer effects through multiple signaling pathways. Notably, the use of caspase inhibitors Z-DEVD-FMK and Z-LEHD-FMK, which specifically inhibit caspase-3 and caspase-9, respectively, can rescue cells from apoptosis induced by FIR treatment.

**Conclusion:**

This study systematically elucidated that FIR exerts anti-tumor effects through multiple mechanisms, including inducing MM cell apoptosis, exacerbating hypoxic stress, and causing cell cycle arrest. The findings provide new insights and approaches for MM treatment and establish a theoretical foundation for the clinical application of FIR in cancer therapy.

**Supplementary Information:**

The online version contains supplementary material available at 10.1186/s12885-025-14031-0.

## Introduction

Melanoma, a malignant tumor originating from melanocytes in the skin, exhibits high lethality and invasiveness [1]. Its etiology is complex, involving factors such as ethnicity, genetic mutations, mole types and quantities, ultraviolet radiation exposure, and immune response. With an incidence rate of approximately 1%, melanoma accounts for around 80% among all skin cancers-related deaths [2], largely due to the limitations of current treatment modalities. Current treatment options for melanoma include surgical excision, chemotherapy, targeted therapy, immunotherapy, and radiotherapy. With the rapid advancement of artificial intelligence, personalized immunotherapy strategies based on mutational genomics have achieved significant breakthroughs, particularly in improving the accuracy of treatment response prediction and therapeutic efficacy [3]. In the exploration of novel treatments, the Ferroptosis Score model, an early research achievement, has shown promising application prospects [4, 5], while the combined application of Photodynamic Therapy (PDT) and Immune Checkpoint Inhibitors (ICIs) has achieved remarkable therapeutic effects through synergistic anti-tumor immune mechanisms [6]. However, these innovative therapies face significant challenges in clinical translation, particularly in cases where surgery is impractical or in instances of large tumors at critical anatomical sites, posing considerable therapeutic difficulties [2]. Consequently, there is an urgent need to develop innovative, effective, and tailored therapeutic approaches to address the formidable challenge of MM.

Addressing the need for new therapeutic strategies, apoptosis, or programmed cell death, has become a significant focus. As the most common form of cell death in organisms, it is characterized by the absence of an inflammatory response. Triggered by specific cellular cues, it initiates a cascade of protein hydrolysis events mediated by the caspase protease family, with caspase-3 acting as the principal executor of apoptosis. Activation of cysteine proteases induces distinct morphological changes associated with apoptosis, including cell shrinkage, DNA fragmentation, chromatin condensation, membrane blebbing, and preservation of cell membrane integrity [7, 8]. This process involves both extrinsic and intrinsic pathways. The extrinsic pathway is initiated by the binding of ligands to cell surface death receptors, culminating in the formation of the death-induced signaling complex, activation of caspase-8, and subsequent initiation of caspase-3 [7]. In contrast, the intrinsic pathway, triggered by mitochondrial stress, includes both caspase-dependent and caspase-independent mechanisms. The caspase-dependent mechanism is marked by apoptosome formation, activation of caspase-9 and caspase-3, and hydrolysis of critical proteins [8], while the latter involves the release of apoptosis-inducing factor (AIF) from mitochondria. Both pathways are associated with a decline in mitochondrial transmembrane potential (MOMP, △Ψm) and are regulated by the expression of Bax and Bcl-2 [9]. 

Among materials with unique properties, graphene, a two-dimensional nanomaterial with a hexagonal crystal structure, has emerged as highly promising for clinical diagnosis and management [10]. Graphene films, when electrified, emit carbon-based far-infrared waves (7–14 μm), wavelength highly compatible with human absorption [11] and capable of penetrating up to 5 centimeters beneath the skin [12]. When far-infrared radiation (FIR) interacts with human or biological systems, its energy is absorbed by water molecules and molecular bonds in tissues, inducing molecular vibrations and rotations that produce a mild radiative heating effect [13]. In cancer treatment, FIR has demonstrated significant potential. *Ting et al.* previously used ceramic-generated far-infrared radiation (cFIR) to suppress nitric oxide (NO) production, reducing the proliferation rate of melanoma cells by 11.8% [14, 15]. Building on these findings, *Hu et al.* further developed a method using graphene-emitted far-infrared waves to significantly impede the proliferation and metastasis of triple-negative breast cancer cells, with no evident adverse effects [11]. These studies provide a new approach for the development of FIR in cancer treatment, although the mechanisms underlying its therapeutic effects on tumors, particularly melanoma, remain incompletely understood.

In this study, we investigated the therapeutic effects of FIR on MM and its underlying mechanisms. FIR significantly induces apoptosis, while hypoxic stress and cell cycle arrest also regulate the proliferation of B16F10 melanoma cells. Further validation in mouse models confirms the significant anti-tumor efficacy of FIR. In conclusion, our study highlights FIR-induced apoptosis as an innovative and promising therapeutic strategy, laying the experimental foundation for developing FIR-based targeted anti-cancer drugs and demonstrating significant potential for clinical translation.

## Methods

### Material characterization

Samples were characterized by scanning electron microscopy (GeminiSEM 500, 10 kV), transmission electron microscopy (JEOL JEM-2100 plus) and Raman spectroscopy (HORIBA XploRA, 532 nm laser excitation, 50× objective). Graphene films were purchased from Xiamen XiHe Technology Co., Ltd. (Number: XH-GHF-07; core size: 4 × 8 cm^2^), and their preparation process was shown in our previous report [16]. 

### Preparation and characterization of graphene electrothermal films

Graphene conductive ink was prepared and screen-printed onto a flexible polyimide (PI) film, with silver paste used as the electrode. The typical preparation of graphene films involves screen-printing graphene conductive ink onto a PI sheet, curing it at 150℃ for 30 min, then screen-printing silver paste on both ends as electrodes, and attaching copper strips to the electrodes. Finally, a laminating machine is used to apply centrifuge film for subsequent testing. The prepared graphene films were analyzed using transmission electron microscopy (TEM) and scanning electron microscopy (SEM). The analysis revealed the presence of graphene flakes within the films (Fig. S1a and b), with Raman spectroscopy confirming the existence of very thin graphene material through a characteristic peak at 2716.8 cm^− 1^ (Fig. S1c). Multiple heating tests (Fig. S1d) and stability assessments of film uniformity (Fig. S1e) demonstrated graphene’s excellent thermal stability, ensuring reproducibility in therapeutic experiments.

### Emission spectroscopy detection

The emission spectrum was collected by the Wuhan Product Quality Supervision and Inspection Institute using a self-built spectrometer with a response range of 2.5–150 μm. A blackbody radiation source was used as the reference and calibration source. The graphene film emitter was passively heated by a heater with a temperature fluctuation of less than 1%. Fourier transform infrared spectroscopy was used to collect the FIR data.

### Measurement of electric thermal radiation conversion

The test material is driven by a DC power supply under ambient conditions. Temperature is measured using an infrared camera, and the actual temperature is determined by setting the emissivity value. Thermal radiation power can be calculated using the Stefan-Boltzmann law, E = ε S σ(T4 − T0 4), where E is the radiation power, ε is the emissivity, σ is the Stefan constant (5.67 × 10^− 8^ W m^− 2^ K^− 4^), S is the surface area of the electrothermal material, T is the temperature of the electrothermal material, and T0 is the ambient temperature. By simultaneously monitoring the input power, the thermal radiation efficiency of the electrothermal material at the operating temperature can be calculated as η = E/P, where η is the thermal radiation efficiency of the electrothermal material at the operating temperature, and P is the input power.

### Cell culture and treatment

B16F10 cells, derived from C57BL/6J mouse melanoma, exhibit a spindle-shaped, epithelial-like morphology. For in vitro safety assessment, human skin fibroblasts (HFF) and immortalized human epidermal cells (HaCaT) were used. HaCaT cells were purchased from the China Center for Type Culture Collection, while B16F10 cells and HFF were obtained from the National Collection of Authenticated Cell Cultures. All cells were cultured in a humidified atmosphere at 37℃ with 5% CO_2_ and saturated humidity. After 24 h of cell plating, the culture medium was replaced, and graphene was placed beneath the Petri dish and exposed to FIR at 32.8 mW/cm² for 45 min. FIR cytotoxicity was assessed at regular intervals. Since B16F10 cell viability decreased to approximately 25% at 24 h post-FIR intervention, and microscopic observation revealed that most tumor cells were floating and dead, this time point was selected as the final sample collection time for subsequent experiments.

### Mouse grouping and treatment

The experiment, approved by Xiamen University’s Animal Care and Use Committee (approval number XMULAC20190070), used twenty 8-week-old female C57BL/6J mice divided into Control, Model, and FIR groups. Mice received an intradermal injection of B16F10 cells (5 × 10^6^/mL). On the third day, FIR treatment at 32.8 mW/cm^2^ for 45 min was initiated. Daily monitoring of tumor growth showed that within 5 days, tumors in the model group reached approximately 400 mm^3^. Discontinue treatment and administer isoflurane (RWD, CHN) inhalation anesthesia (1–3% in oxygen) to render the mouse unconscious. Photographs of the mice were taken for documentation, followed by ocular blood collection. Perform cervical dislocation to euthanize the mice instantaneously, minimizing pain and distress in compliance with ethical guidelines.

### Cell proliferation assay

B16F10 cells in the logarithmic growth phase were seeded into a 96-well plate at a density of 4,000 cells per well and allowed to adhere for 24 h. After replacing the culture medium, the cells were pretreated with Z-DEVD-FMK (200 µM, APExBIO, USA) or Z-LEHD-FMK (20 µM, APExBIO, USA) for 3 h. The cells were then exposed to FIR intervention. At 24 h post-irradiation, 10 µL of CCK-8 reagent (Biosharp, CHN) was added to each well, and the plate was incubated at 37 °C in the dark for 1 h. Absorbance at 450 nm was measured using a multifunctional microplate reader.

B16F10 cells were seeded into a 96-well plate and cultured for 24 h before FIR intervention. At 24 h post-FIR treatment, the reaction substrate mixture was prepared according to the manufacturer’s instructions for the LDH assay kit (Promega, USA). Then, 50 µL of culture supernatant from each well was transferred to a new 96-well plate, and 50 µL of the pre-mixed reaction solution was added to each well. The plate was incubated at room temperature in the dark for 30 min. Absorbance at 490 nm was measured using a multifunctional microplate reader, and the LDH release rate was calculated to assess cell membrane integrity.

### RNA sequencing and differential expression analysis

B16F10 cells and tumor tissue were treatment with FIR. Total RNA was extracted using TRIzol reagent (Invitrogen, USA) and submitted to Chidio Biotechnology Co., Ltd. for RNA sequencing (RNA-seq) and differential expression analysis. Heatmap generation, Kyoto Encyclopedia of Genes and Genomes (KEGG) analysis, and Gene Set Enrichment Analysis (GSEA) analysis were performed using Omicshare.

### Cell apoptosis detection

After cell plating, the cells were collected and resuspended in 1x binding buffer (Life Technologies, CHN). After filtration through a 300-mesh filter screen, Annexin V-FITC (5 µL) and PI (10 µL) were added. Detection was performed immediately using a flow cytometer. After fixation, the treated cells were incubated with proteinase K (Beyotime, CHN) was added and incubated according to the manufacturer’s instructions. Subsequently, 50 µL of TDT enzyme working solution was added. After staining, the nuclei were washed and observed under a fluorescence microscope.

### Detection of caspase enzyme activity

The cell plating and treatment procedures followed the same protocol as described above. After 24 h, the samples were harvested and lysed for protein extraction. The protein concentration was quantified using the Bradford method (Beyotime, CHN). An appropriate amount of four chromogenic substrates was used as reaction substrates, and the enzyme activity reaction system was prepared according to the manufacturer’s instructions for the caspase assay kit (Beyotime, CHN). The reaction system was incubated at 37 °C for 24 h, after which absorbance at 405 nm was immediately measured using a multifunctional microplate reader. The activities of Caspase-3, -8, and − 9 were evaluated based on the cleavage of the chromogenic substrates.

### Mitochondrial membrane potential detection (MOMP, ΔΨm)

The experiment was performed in a 6-well plate, involving cell plating, treatment, and sample collection. After cell counting and centrifugation, the cells were resuspended. An appropriate volume of pre-prepared JC-1 working solution (Solarbio, CHN) was added to the cell suspension and incubated at 37 °C in the dark for 20 min. Following centrifugation, the supernatant was discarded, and the cells were washed twice with 2 mL of 1× buffer. Finally, the cells were resuspended in approximately 400 µL of buffer, filtered through a 300-mesh sieve into flow cytometry tubes, and analyzed by flow cytometry at 2–4 °C.

### ROS detection

B16F10 cells were seeded into confocal dishes and 6-well plates at a density of 2.5 × 10⁵ cells per well and cultured for 24 h, after which they were subjected to FIR intervention. Following the manufacturer’s instructions for the DCFH-DA assay kit (Promega, USA), the probe working solution was prepared by diluting it in serum-free medium at a ratio of 1:1000 to a final working concentration of 5 µM. The DCFH-DA working solution in fresh serum-free medium was added to the confocal dishes and collected cell samples, followed by incubation at 37 °C in the dark for 30 min and subsequent washing steps. Finally, the samples were analyzed using confocal microscopy and flow cytometry.

### Cell cycle assay

Following cell plating, treatment, and sample collection, 1 mL of pre-cooled 70% ethanol was added to fix the cells overnight at 4 °C. The next day, the cells were centrifuged and washed twice with PBS. Subsequently, 100 µL of staining solution containing 100 µg/mL RNase A (Elabscience, CHN) was added, and the cells were incubated in a 37 °C water bath in the dark for 30 min. After centrifugation and washing, 400 µL of 50 µg/mL PI staining solution was added, and the cells were filtered through a 300-mesh sieve before analysis using a flow cytometer.

### Membrane fluidity detection

The 10 mM TMA-DPH stock solution (Bestbio, CHN) was diluted in PBS at a 1:1000 ratio to a final working concentration of 10 µM. Following sample collection and washing, the cells were resuspended in PBS containing the TMA-DPH working solution at a density of 1 × 10⁶ cells/mL and incubated at 37 °C in the dark for 30 min. After washing and centrifugation, the cells were resuspended in 1 mL of PBS, and 400 µL of the suspension was transferred to a cuvette for analysis using a fluorescence spectrophotometer.

### Immunohistofluorescence staining

The tissue underwent dewaxing and antigen retrieval procedures. After fixation, a 1-hour blocking step was performed, followed by overnight incubation at 4 °C with primary antibodies [Bax (ab32503), Bcl-2 (ab32124), cleaved caspase-3 (ab214430), Higd2a (ab135399), HSP70 (ab2787), MLKL (ab184718), pMLKL (ab196436) from Abcam, USA; cleaved caspase-8 (#8592), HSP90 (4877s), Tom 20 (#42406), AIF (#5318), PARP (#9532), HIF-1α (#48085) from Cell Signaling Technology, USA; and MAP1LC3 (sc-271625) from Santa Cruz Biotechnology, CHN]. After thorough washing, secondary antibodies [Alexa Fluor^®^ 488 AffiniPure Donkey Anti-Rabbit IgG and Cy3 AffiniPure Donkey Anti-Rabbit IgG] were applied for 2 h. Finally, scanning was conducted using a panoramic digital slide optical fiber system.

### Western blotting (WB)

The proteins were extracted, quantified using BSA method, electrophoresed, and subsequently transferred to a polyvinylidene fluoride (PVDF) membrane (Invitrogen, CHN). After blocking for 1 h, the membranes were incubated overnight with primary antibodies (as mentioned above). Following thorough washing steps, secondary antibodies from Li-Cor, USA (IRDye 680RD Donkey Anti-Mouse and IRDye 800CW Donkey Anti-Rabbit) were applied and incubated. Protein band quantification was performed using the Li-Cor Odyssey Infrared Imaging System.

### Statistical analysis

Each experiment was repeated at least three times to ensure reliability. Data are expressed as the mean ± SEM (standard error of the mean) and were analyzed using Prism 8 (GraphPad, San Diego, CA). Statistical comparisons were conducted using t-tests or one-way analysis of variance (ANOVA). A significance level of *P* < 0.05 was applied to all analyses.

## Results

### Time-Dependent Inhibition of B16F10 cell proliferation by FIR

**Fig. 1:**
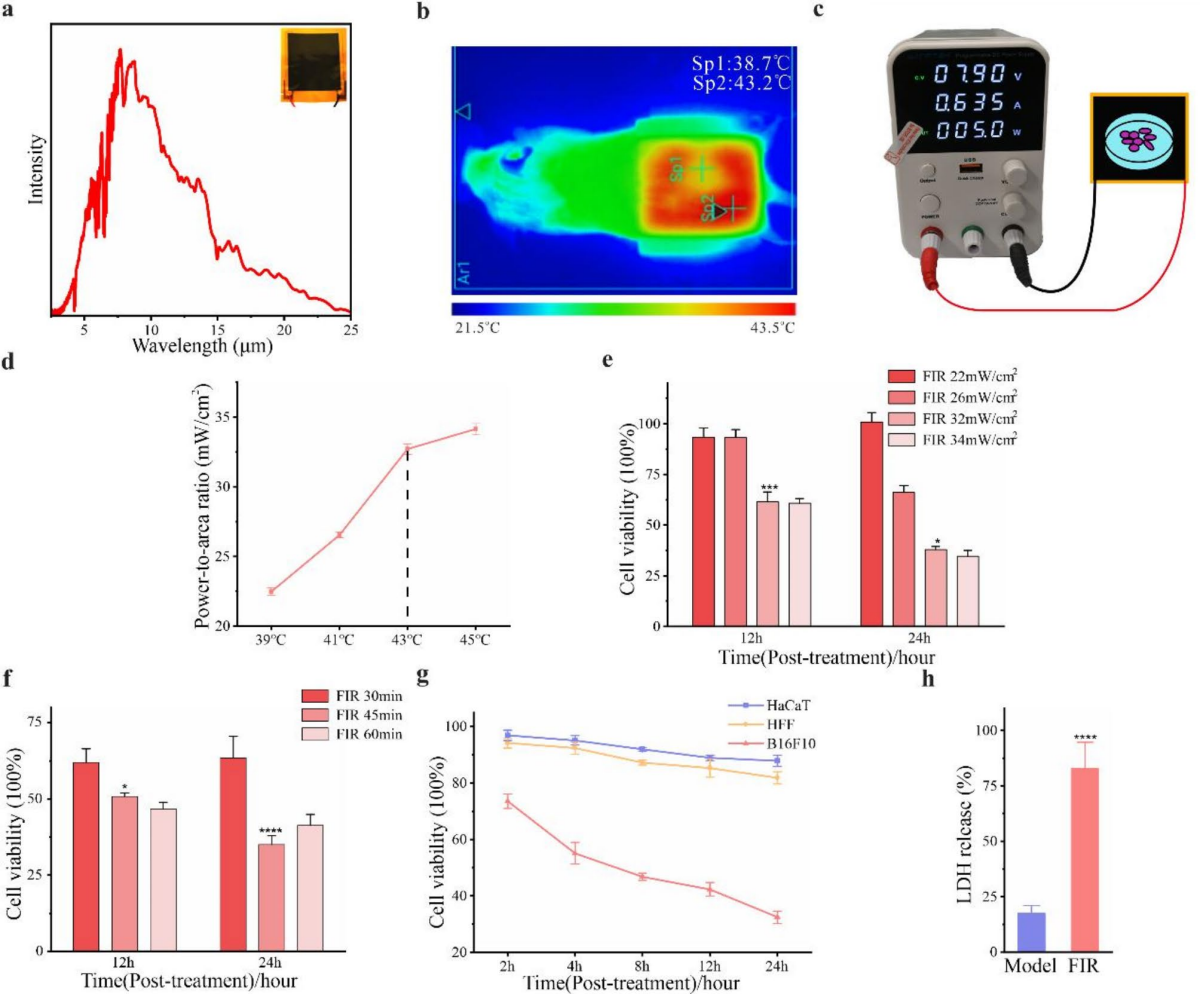
Inhibition of B16F10 Cell Proliferation by FIR in vitro. **a)** Comparison of surface emissivity among graphene films. **b-c)** Schematic diagram of the FIR device and its therapeutic illustration in mice and cells. **d)** Power density ratios of FIR at different temperatures. **e)** Proliferation activity of B16F10 cells under various conditions after FIR treatment. **f)** Activity of B16F10 cells treated with FIR at 32.8 mW/cm^2^ for various durations. **g)** Cell activity measured after FIR treatment at 32.8 mW/cm^2^ for 45 min. **h)** Measurement of LDH activity. Data are presented as the mean ± SD of three independent experiments, with significance levels denoted as *P < 0.05, **P < 0.01, ***P < 0.001, and ****P < 0.0001

In this experiment, the graphene film used emits FIR in the wavelength range of 2.5 to 25 micrometers when electrified (Fig. 1a). Concurrently, experimental methods for graphene-based cell therapy and mouse therapy were developed (Fig. 1b and c).

To assess the impact of FIR on B16F10 cell proliferation, the measurable variables were defined as power density and exposure time. The power density levels at 39 °C, 41 °C, 43 °C, and 45 °C were measured (Fig. 1d) to identify optimal treatment conditions. The selected conditions were then applied at various power densities for 30 min, 45 min, and 60 min at equivalent temperatures to determine the optimal treatment duration for assessing cell viability.

We observed a notable decrease in B16F10 cell proliferation activity at 32.8 mW/cm^2^ (43 °C) and 34.2 mW/cm^2^ (45 °C) after 12 and 24 h of FIR treatment. However, the difference in cell viability between these two conditions was not statistically significant (*P* = 0.1428, Fig. 1e). Therefore, 32.8 mW/cm^2^ was selected as the optimal treatment condition. Cells treated with 32.8 mW/cm^2^ for varying durations exhibited the most significant decrease in cell viability after 45 min (Fig. 1f). Subsequent experiments using this treatment condition on B16F10 cells showed a reduction in cell proliferation activity of 67.55 ± 1.58% in the FIR group (Fig. 1g), and with cell proliferation activity decreasing as post-treatment time increased. Simultaneously, the release of LDH increased, indicating cell membrane rupture (Fig. 1h). These findings suggest that FIR treatment at 32.8 mW/cm^2^ for 45 min effectively reduces the survival rate of B16F10 cells. To systematically evaluate the biosafety of FIR, HFF and HaCaT cells were also investigated. Experimental results demonstrated that 24 h after FIR exposure, the proliferation rates of HFF and HaCaT cells remained at 81.8% ± 3.1% and 84.7% ± 2.6%, respectively (Fig. 1g). These findings indicate that FIR exerts minimal inhibitory effects on the growth of normal epidermal cells. Thus, under the FIR parameters that effectively inhibit melanoma growth, the risk of damage to normal epidermal cells is negligible.

### Transcriptomic analysis of FIR effects on melanoma cells via RNA-seq

**Fig. 2:**
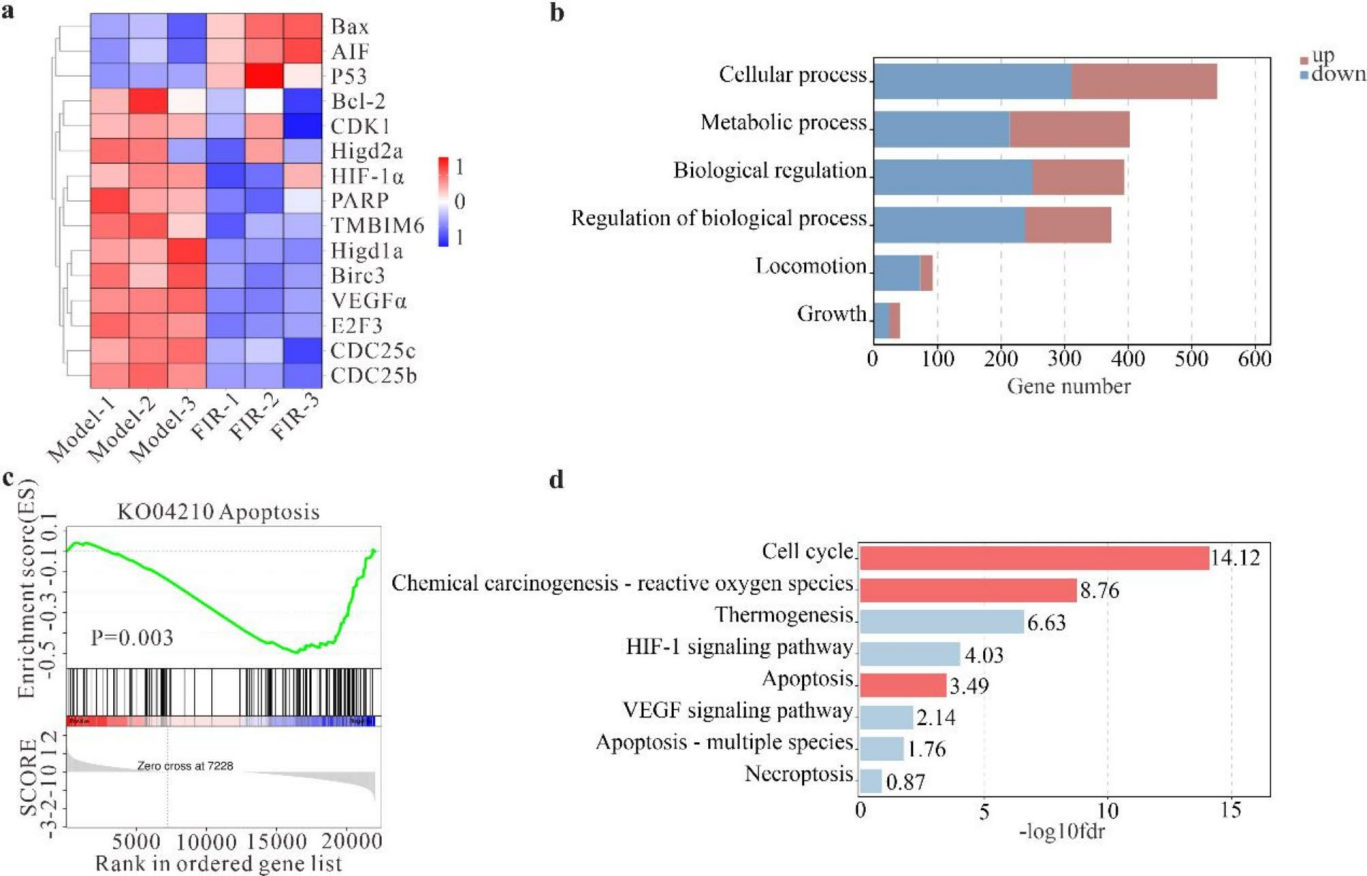
RNA sequencing analysis of the impact of FIR on the expression profile of B16F10 cells. **a)** Heatmap of differentially expressed genes in the transcriptome of B16F10 cells following FIR treatment, generated through differential expression analysis. **b-d)** Results of GO, GSEA, and KEGG analyses, showing the enrichment of significant pathways in FIR-treated tumor cells

To explore the molecular mechanism underlying FIR-induced melanoma cell death, we conducted systematic gene expression analysis using RNA-seq technology. A heatmap was used to analyze gene sets that exhibited significantly altered expression levels in tumor cells after FIR treatment (Fig. 2a). The upregulation of pro-apoptotic genes such as Bax, p53, and PARP, alongside downregulation of anti-apoptotic genes like Bcl-2, Birc, and Tmbim6, suggests that FIR may activate the apoptotic pathway in tumor cells. Furthermore, we noted inhibition of hypoxia-related genes such as Higd2a, VEGFα, and HIF-1α, indicating that FIR intervention may diminish the hypoxia adaptability of MM cells. The expression of cell cycle regulatory genes such as CDK1, E2F, and CDC15 also showed a downward trend, suggesting that FIR may inhibit tumor cell proliferation by interfering with the cell cycle process. Additionally, GO analysis revealed that FIR intervention may negatively affect the metabolism, biological regulation, and proliferation of tumor cells (Fig. 2b). Simultaneously, KEGG and GSEA enrichment analyses underscored the significant impact of FIR treatment on signaling pathways essential for regulating cell proliferation and death, including apoptosis, cell cycle, and hypoxic stress (Fig. 2c and d). These findings collectively suggest that FIR treatment induces cell cycle arrest, apoptosis, and hypoxic stress at the transcriptome level, providing insights into the underlying mechanisms of FIR-induced cell death.

### Implication of apoptosis in FIR-Induced B16F10 cell responses

**Fig. 3:**
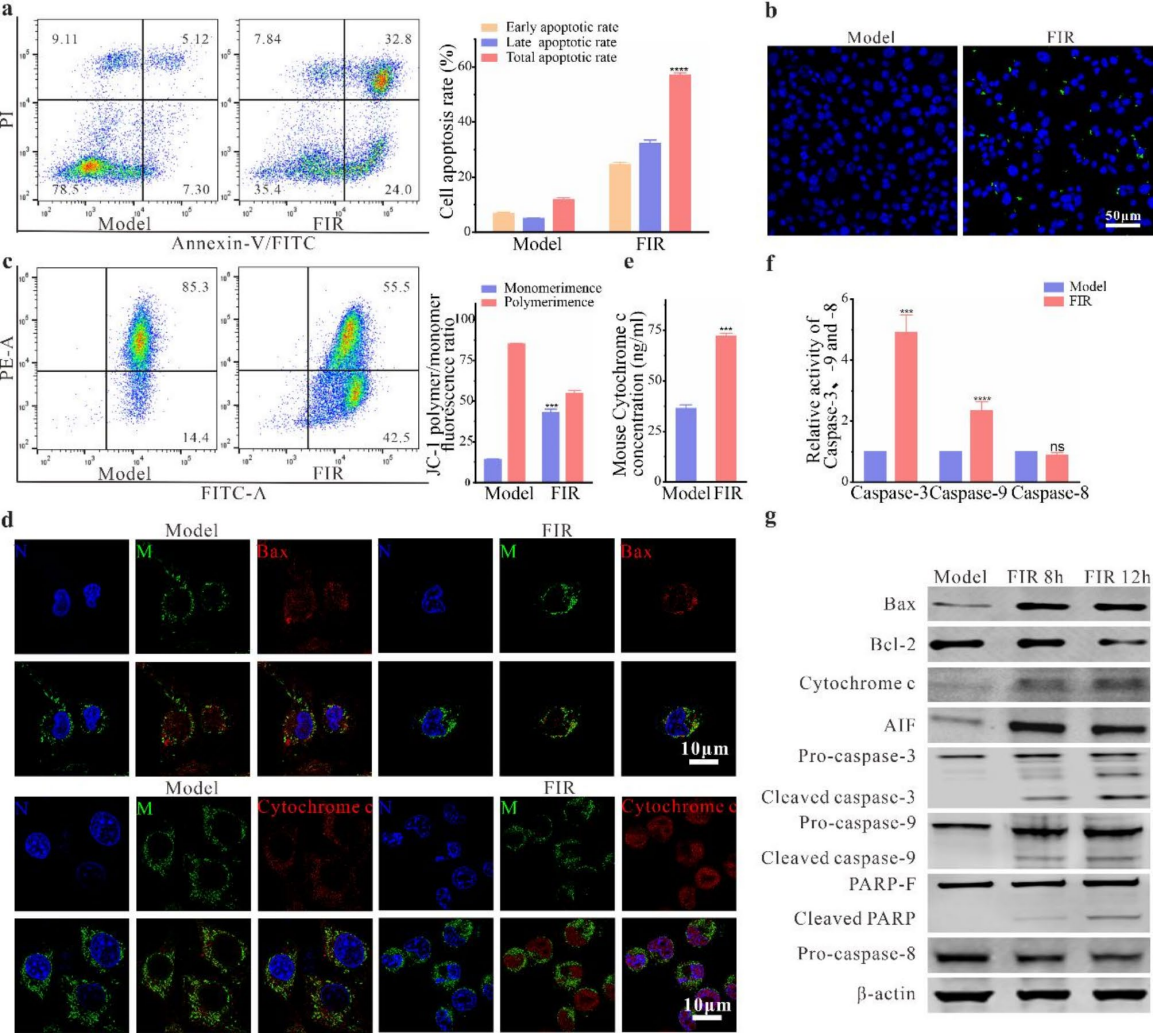
Effects of FIR treatment on apoptosis and mitochondrial changes in B16F10 cells. **(a)** Images of B16F10 cells stained with Annexin V-FITC and PI to detect apoptosis. **(b)** TUNEL staining images showing apoptotic cells with positive staining in B16F10 cells. **(c)** Flow cytometry results showing changes in MOMP and quantification of the JC-1 polymer-to-monomer ratio in B16F10 cells. **(d)** Immunofluorescence staining images showing the localization changes of cytochrome c and Bax in B16F10 cells. N: nucleus; M: mitochondria. **(e)** Cytosolic cytochrome c levels determined by ELISA. **(f)** Activity evaluation of Caspase-3, Caspase-8 and Caspase-9 in B16F10 cells at 24 h after FIR treatment. **(g)** Western blot analysis at 8 h and 12 h after FIR treatment to detect protein expression levels. Data are presented as the mean ± SD of three independent experiments, with significance levels denoted as *P < 0.05, **P < 0.01, ***P < 0.001, and ****P < 0.0001

Our analysis demonstrated that the apoptosis rate of B16F10 cells after FIR treatments was 56.8 ± 3.3% (Fig. 3a). Meanwhile, confocal microscopy revealed an increase in TUNEL-staining positive cells following FIR treatment (Fig. 3b). These findings collectively support the notion that FIR can induce apoptosis in B16F10 cells. Subsequently, flow cytometry results indicated that a significant decrease in the ratio of JC-1 polymers to JC-1 monomers after FIR treatment (Fig. 3c), suggesting a reduction in MOMP, which may lead to the leakage of mitochondrial contents into the cytoplasm. Immunofluorescence staining revealed the disruption of the normal tubular mitochondrial network in B16F10 cells, accompanied by significant translocation of cytochrome c and Bax from mitochondria to the cytoplasm (Fig. 3d), resulting in a marked increase in cytoplasmic cytochrome c content (Fig. 3e). Based on these observations, we hypothesize that FIR amplifies the activation of the apoptotic pathway and promotes the formation of apoptotic bodies by diminishing the mitochondrial membrane potential of B16F10 cells and facilitating the release of cytochrome c and Bax into the cytosol through enhanced mitochondrial permeability.

Apoptosis is categorized into intrinsic and extrinsic pathways. To investigate these pathways, we assessed the levels of caspase-3, -8, and − 9 in B16F10 cells. Our findings revealed a significant increase in the activity of caspase-3 and caspase-9 in FIR-treated cells compared to untreated cells, whereas the activity of caspase-8, a critical enzyme in the extrinsic apoptosis pathway, remained unchanged (Fig. 3f). Furthermore, western blot analysis revealed upregulation of Bax and cytochrome c — key proteins in the intrinsic apoptosis pathway — in the FIR-treated group, along with the presence of cleaved fragments of caspase-3 and − 9 (Fig. 3g). Concurrently, caspase-3 activation resulted in the cleavage of PARP, yielding an 89 kDa fragment. Intriguingly, AIF, a caspase-independent apoptosis inducer, exhibited increased leakage from the mitochondria. These findings collectively suggest that FIR markedly enhances caspase-3 activation, thereby inducing intrinsic apoptosis.

**Fig. 4:**
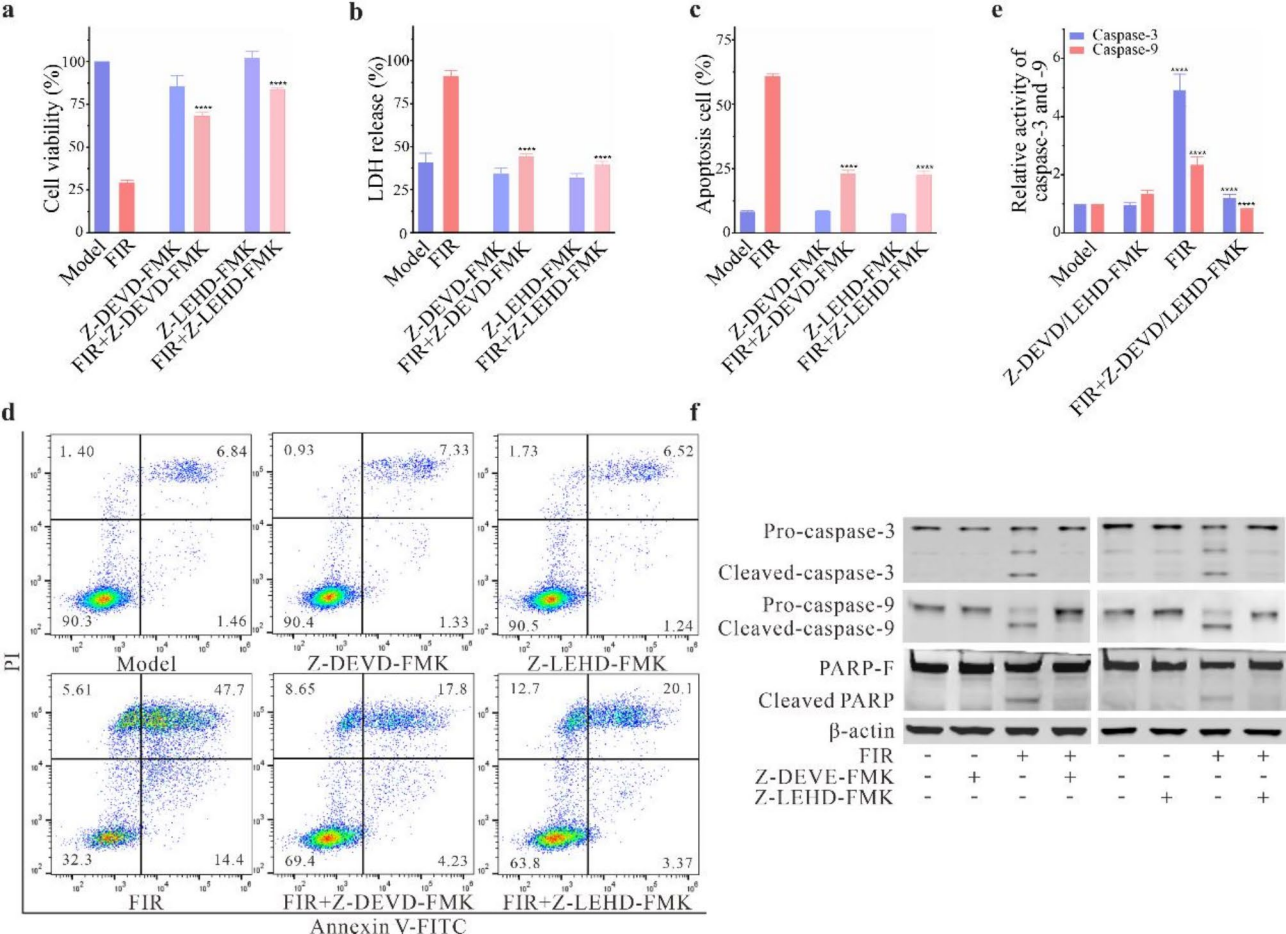
Effect of caspase inhibitors on caspase activity induced by FIR in B16F10 cells. **(a)** CCK-8 assay results showing the proliferation activity of B16F10 cells after treatment with caspase inhibitors. **(b)** Measurement of LDH activity. **c-d)** Detection of apoptotic B16F10 cells stained with Annexin V-FITC and PI. **e)** Relative activity of Caspase-3 and Caspase-9. **f)** Protein expression levels of Caspase-3, Caspase-9, and cleaved PARP. Data are presented as the mean ± SD of three independent experiments, with significance levels denoted as *P < 0.05, **P < 0.01, ***P < 0.001, and ****P < 0.0001

To further validate the involvement of caspases in FIR-induced apoptosis, we employed two caspase inhibitors: the caspase-3 inhibitor Z-DEVD-FMK and the caspase-9 inhibitor Z-LEHD-FMK. Treatment with these inhibitors rescued the majority of dying cells and enhanced the proliferative viability of B16F10 cells (Fig. 4a and b). Flow cytometry results demonstrated a significant reduction in the percentage of apoptotic cells (Fig. 4c and d). Moreover, in the intrinsic apoptotic pathway, the activation of caspase-3 and caspase-9, as well as the cleavage of PARP induced by FIR, was notably suppressed by the caspase-3 and/or caspase-9 inhibitor (Fig. 4e and f). These findings suggest that FIR-induced apoptosis in B16F10 cells is mediated by the mitochondria-dependent caspase-3 and caspase-9 pathway, with caspase-3 potentially playing a more crucial role.

### Significant suppression of tumor growth by FIR in C57BL/6J tumor-bearing mice

**Fig. 5:**
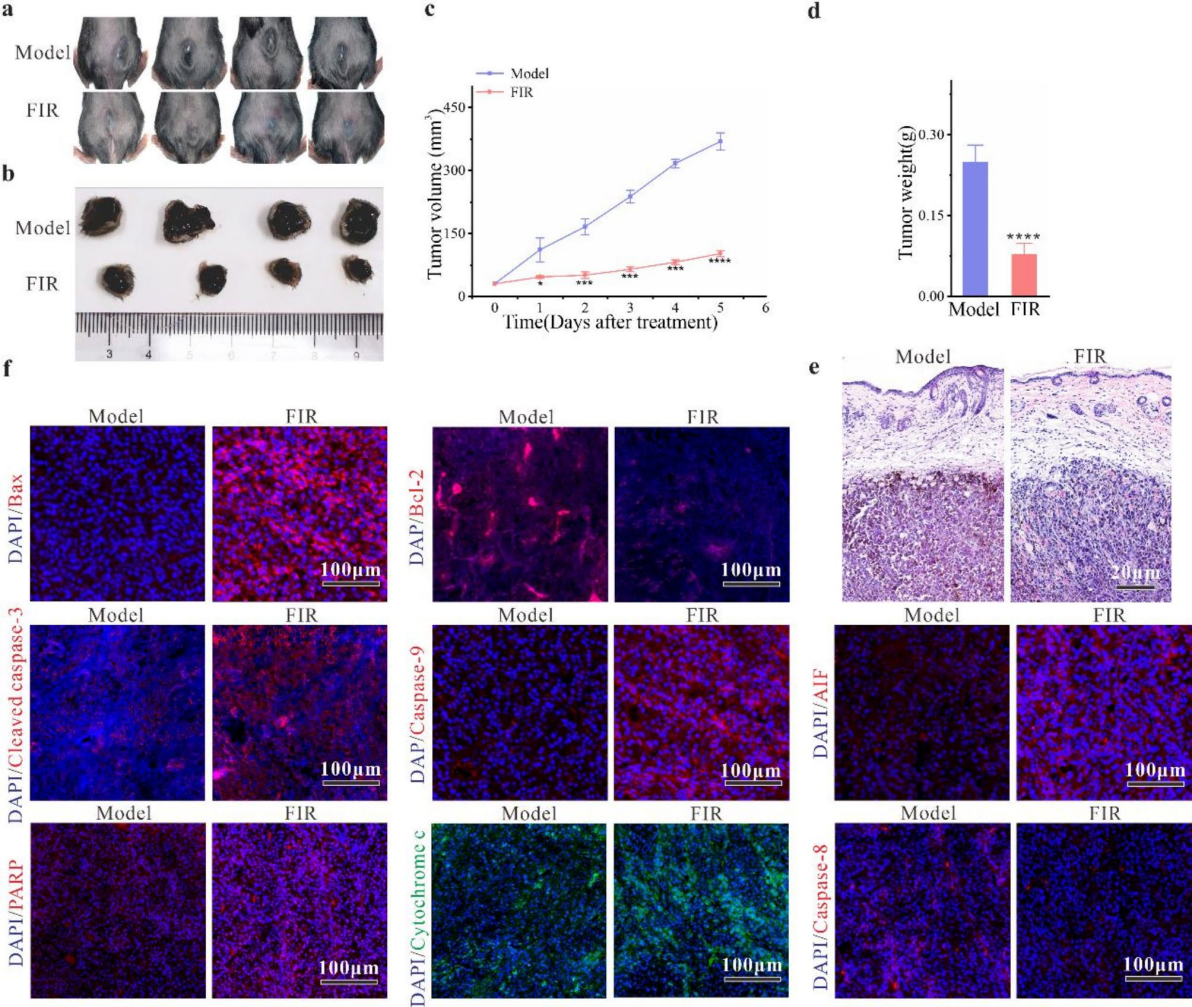
In vivo tumor suppression by FIR in C57BL/6J mice. **a-b)** Tumor images taken 6 days post-treatment. **c-d)** Melanoma tumor volume and weight measurements. **e)** Hematoxylin and eosin (H&E) staining of tumor tissues. **f)** Immunofluorescence staining of tumor tissues. Data are presented as the mean ± SD of three independent experiments, with significance levels denoted as *P < 0.05, **P < 0.01, ***P < 0.001, and ****P < 0.0001

Our in vitro findings prompted an investigation into the efficacy of FIR in C57BL/6J tumor-bearing mice. Remarkably, tumor volume and weight were significantly reduced in the FIR group compared to the model group (31.61%, *P* < 0.05) (Fig. 5a-d), with an inhibition rate as high as 73%. Histological examination via H&E staining revealed that tumors in the FIR group exhibited a denser texture, reduced cellular atypia, and a more regular cellular arrangement compared to the model group (Fig. 5e). As is well known, the predominant component of biological fluids is water, and far-infrared energy is sufficient to induce molecular rotation and vibration of bonds (including water molecules), resonating at the same frequency as cells [12]. Therefore, we hypothesize that this effect may be attributed to the resonance phenomenon induced by the alignment between FIR and the absorption peak of biological tissues. Additionally, an assessment of the safety of FIR treatment revealed no morphological changes in vital organs (spleen, lung, liver, kidney) of tumor-bearing mice, as indicated by H&E staining results (Fig. S2).

To verify whether FIR inhibits tumor growth by inducing apoptosis in vivo, immunohistofluorescence staining was performed on tumor tissues. The results revealed a significant increase in the expression of Bax, cleaved caspase-3, caspase-9, AIF, and PARP, along with a decrease in Bcl-2 expression compared to the model group (Fig. 5f). These findings are consistent with our in vitro experiments, confirming that FIR induces apoptosis in C57BL/6J tumor-bearing mice.

### Induction of HIF-1α-Mediated hypoxic stress in B16F10 cells by FIR

**Fig. 6:**
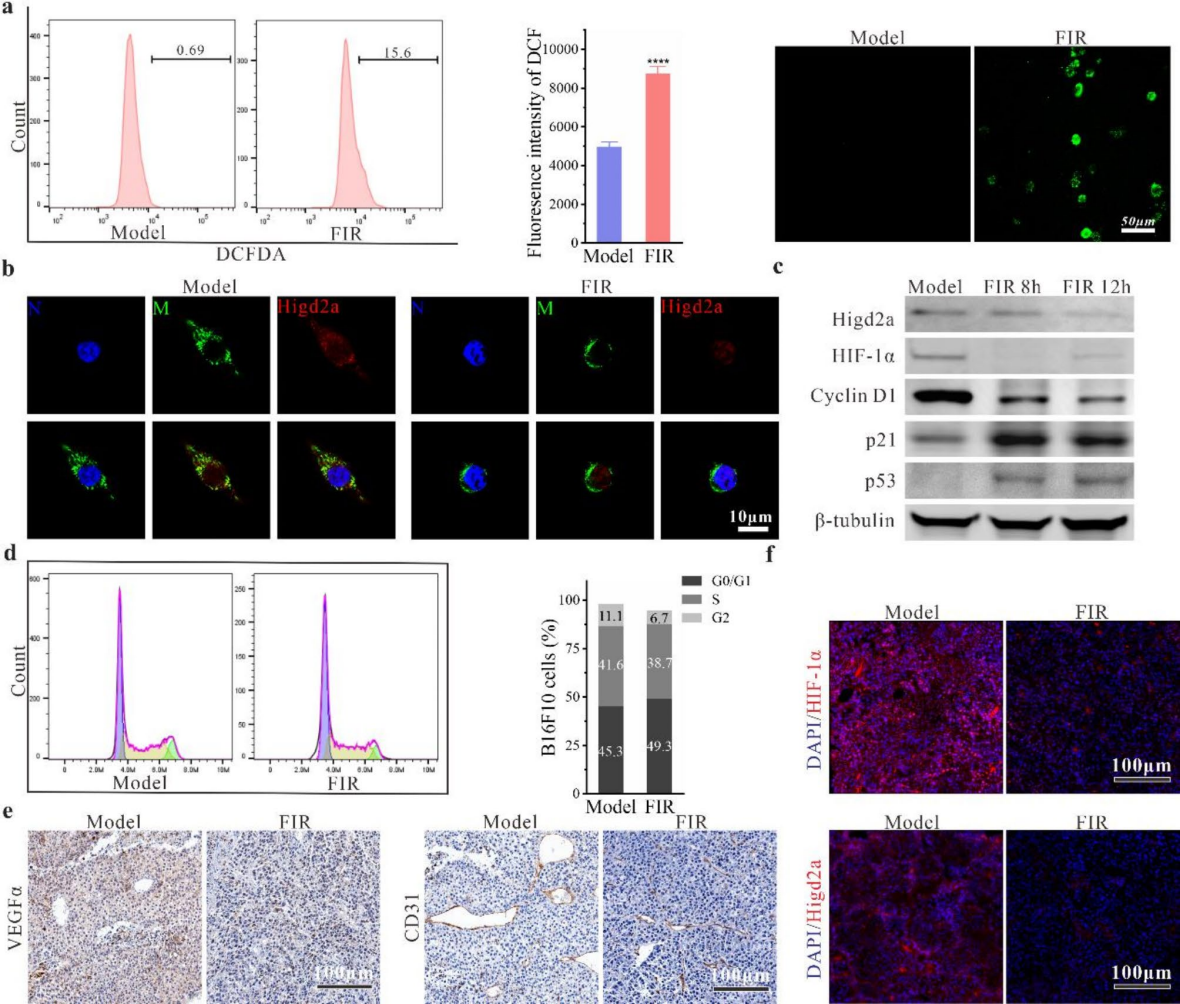
FIR induces hypoxic stress in tumor tissues and cells. **(a)** ROS levels detected by immunofluorescence staining and flow cytometry. **(b)** Immunocytochemistry images showing the intracellular localization of Higd2a. N: nucleus; M: mitochondria. **(c)** Protein expression levels in B16F10 cells determined by Western blot analysis. **(d)** Cell cycle distribution analyzed by flow cytometry. **(e)** Expression of CD31 and VEGFα in tumor tissues detected by immunohistochemical staining. **(f)** Expression levels of Higd2a and HIF-1α in tumor tissues detected by immunofluorescence staining. Data are presented as the mean ± SD of three independent experiments, with significance levels denoted as *P < 0.05, **P < 0.01, ***P < 0.001, and ****P < 0.0001

Heat stress is known to elevate mitochondrial superoxide anions [17] and reduce the activity of superoxide dismutase 1 (SOD-1), leading to increased ROS production [18]. Flow cytometry and immunofluorescence staining were used to assess ROS production after FIR treatment, revealing a significant increase in ROS in B16F10 cells (Fig. 6a), suggesting an early apoptotic event induced by FIR [19]. ROS generation is regulated by respiratory supercomplexes [20–25]. For instance, RCF1, which regulates mitochondrial complexes III and IV in yeast and promotes oxidative phosphorylation (OXPHOS), has orthologs in mice, Higd1a and Higd2a. Previous studies have shown that the absence of RCF1 enhances mitochondrial oxidative stress, resulting in elevated ROS and reduced mitochondrial membrane potential [26]. Higd2a, located in the inner mitochondrial membrane, contains a hypoxia-inducible domain 2 A [27] and translocate to the cell nucleus under specific hypoxic conditions [28]. Following FIR treatment, we observed the translocation of Higd2a from the mitochondria to the cell nucleus in B16F10 cells (Fig. 6b), accompanied by a significant decrease in its expression (Fig. 6c). Moreover, the downregulation of hypoxia-inducible factor (HIF-1α) transcription suggested irreversible hypoxia in B16F10 cells. Hypoxia subsequently triggers the DNA damage response and other stress pathways, activating the tumor suppressor gene p53 and inducing the expression of the p21 protein. Subsequently, p21 binds to the CyclinD1/CDK4 complex and inhibits its activity, leading to cell cycle arrest at the G1 phase (Fig. 6c and d). Immunohistochemical staining of tumor tissues revealed a notable reduction in tumor blood vessels, accompanied by decreased expression of the angiogenesis-inducing factor VEGFα (Fig. 6e), as well as diminished levels of Higd2a and HIF-1α (Fig. 6f). These findings suggest that FIR enhances its anti-tumor efficacy by disrupting tumor blood supply, thereby creating a hypoxic microenvironment.

## Discussion

Currently, melanoma treatment options include surgical resection, chemotherapy, targeted therapy, immunotherapy, and radiation therapy. However, these methods often have limitations in clinical efficacy, resulting in suboptimal outcomes [1, 2]. Consequently, there is growing interest in developing non-invasive therapeutic approaches that can safely eliminate tumors with minimal off-target effects. Inspired by previous research by *K. Hu* and colleagues, which demonstrated the inhibitory effects of graphene far-infrared irradiation on MDA-MB-231 breast cancer, *LoVo* colon cancer, and PC3 prostate cancer, FIR presents a promising outlook for clinical diagnosis and treatment [11, 29–34]. Although some studies have indicated that FIR inhibit tumor cell proliferation and metastasis by reducing NO levels [14, 15, 35–38], the mechanisms underlying FIR’s effects on specific cancer cells remain poorly understood and warrant further investigation. Our study demonstrates that FIR inhibits B16F10 cell proliferation by inducing apoptosis, cell cycle arrest, and hypoxic stress, with significant involvement of the caspase-3 and caspase-9-dependent apoptosis pathway.

In this study, we observed a decline in the proliferative activity of B16F10 cells over time following FIR treatment, consistent with the findings of Mantso et al.., who reported a reduction in cell proliferation after hyperthermia treatment or far-infrared therapy [39]. Meanwhile, recent studies by Zhou et al. have demonstrated that external application of FIR can enhance the production of IFN-γ and increase the membrane fluidity of T cells through a significant resonance-induced thermal radiation effect, thereby promoting TCR aggregation and T cell activation [16]. After FIR intervention, we observed a decrease in membrane fluidity (Fig. S3a) and an increase in the expression of HSP70 and HSP90 (Fig. S3b and c). These findings suggest potential beneficial effects on antigen expression in tumor cells, as well as on T cell migration, dendritic cell uptake, and antigen delivery in MM mice [16, 40]. 

When cells are exposed to radiation, drugs, or other stimuli, cell cycle progression may be halted, reducing the likelihood of tumor cells undergoing self-repair and making them more susceptible to apoptosis signals, often leading to cell death [41]. The Bcl-2 family plays a crucial role in regulating the mitochondrial apoptosis pathway, where the relative expression levels of Bcl-2 and Bax—represented by the Bax/Bcl-2 ratio—are key factors determining cellular susceptibility to apoptotic stimuli [42, 43]. The release of cytochrome c from mitochondria is a pivotal event in apoptosis, and this process is regulated by the balance between pro-apoptotic and anti-apoptotic Bcl-2 family proteins in the mitochondrial outer membrane [44]. When the Bax/Bcl-2 ratio becomes imbalanced in favor of cell death, cytochrome c is released into the cytoplasm, where it binds to Apaf-1 (apoptotic protease-activating factor 1) and procaspase-9 [44], leading to the activation of caspase-9 and caspase-3 and ultimately inducing apoptosis. In contrast, melanocytes with a Bax/Bcl-2 ratio below 1.00 exhibit significant resistance to CD95-induced apoptosis [43]. In tumors, an imbalance in the Bax/Bcl-2 ratio (e.g., overexpression of Bcl-2) can confer resistance to apoptotic stimuli such as chemotherapy and radiotherapy, thereby promoting tumor initiation and progression [45, 46]. In this study, western blot analysis revealed that FIR treatment significantly increased the Bax/Bcl-2 ratio, while cytochrome c, p53, p21, caspase-3, and caspase-9 were significantly induced or activated, and Cyclin D1 was downregulated. Furthermore, DNA damage, along with PARP cleavage and inactivation, accelerated tumor cell instability, making tumor cells more sensitive to DNA damage and triggering apoptosis, as confirmed by Annexin V-FITC/PI staining and TUNEL assays.

Nonetheless, previous observations suggest that heat stimulation might prompt tumor cell necrosis [47]. However, our subsequent analysis of cells and tissues treated with FIR revealed no evidence of the necrotic marker pMLKL, which was further corroborated by RNA-seq data indicating the absence of necrosis (Fig. S3b-d). Interestingly, we discovered that FIR triggers a synergistic cell death mechanism involving both autophagy and apoptosis in melanoma cells by increasing intracellular ROS levels and decreasing mitochondrial membrane potential. Damaged mitochondria expose Tom20 protein on their surface, which acts as an autophagy adaptor [48], binding to LC3bII to mediate selective mitophagy and enhancing autophagic flux through the V-ATPase-ATG16L1-LC3 axis [49]. Research confirmed that FIR treatment significantly upregulates the expression of LC3bII and Tom20 (Fig. S4a and b). This synergistic effect not only clears dysfunctional mitochondria but also simultaneously activates cytochrome c release through Tom20 ubiquitination, creating a pro-apoptotic microenvironment [50]. Notably, the basal level of autophagy is relatively low in early-stage melanoma [51], and FIR can restore autophagic flux to remodel the tumor microenvironment, thereby reversing the oncogenic phenotype. Therefore, we speculate that this dual mechanism—Tom20-mediated mitophagy to clear damaged mitochondria and LC3bII-driven excessive autophagic cell death—provides a molecular basis for FIR-induced killing of melanoma cells.

**Fig. 7:**
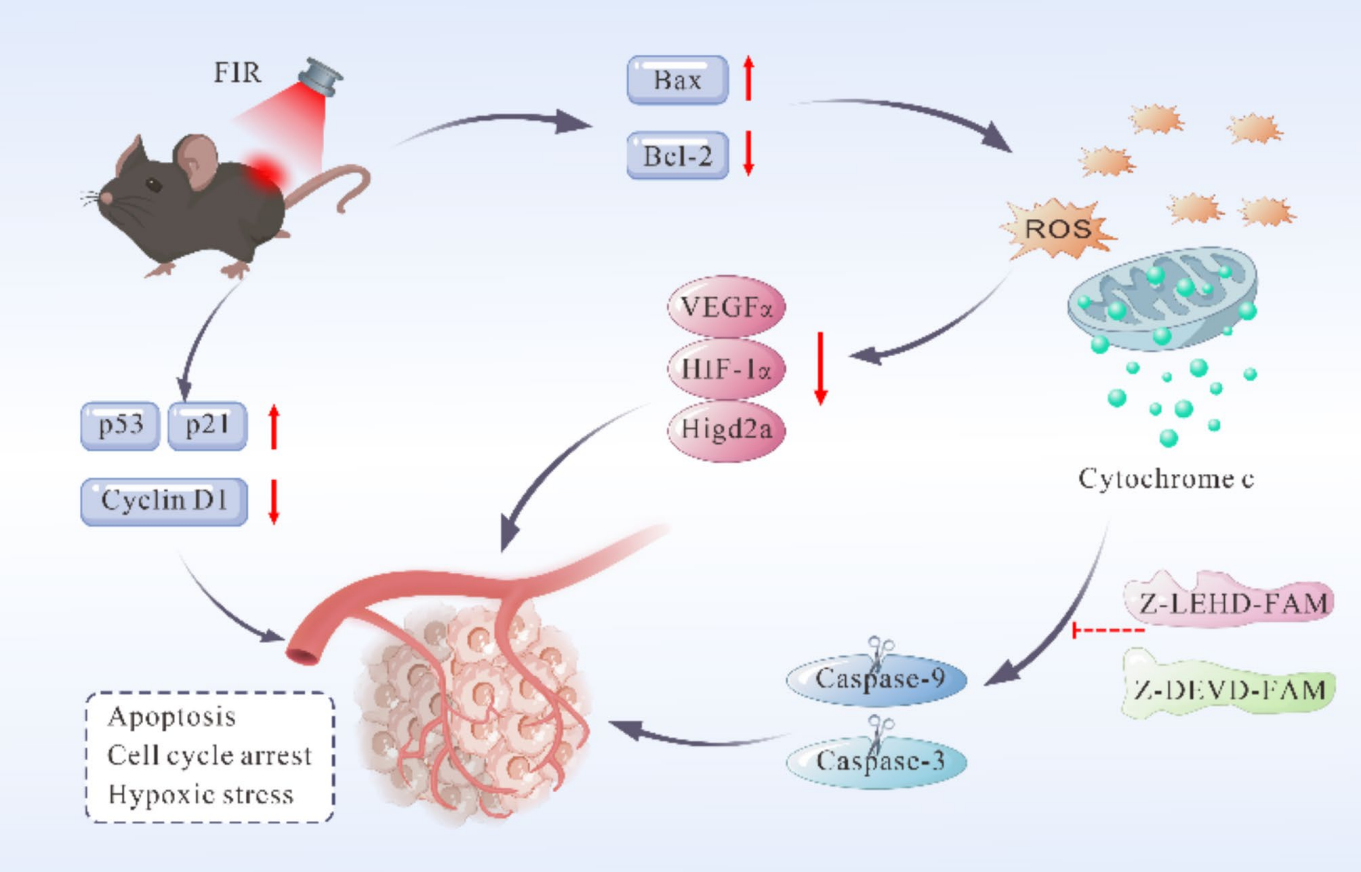
Mechanism diagram of FIR inhibition in MM. FIR intervention in MM upregulates the Bax/Bcl-2 ratio, reduces mitochondrial membrane potential, and increases mitochondrial membrane permeability, promoting the release of cytochrome C into the cytoplasm. There, cytochrome C forms the apoptosome, which activates and cleaves caspase-9 and caspase-3, leading to PARP cleavage and apoptosis. This process can be inhibited by Z-LEHD-FAM and Z-DEVD-FAM. Simultaneously, FIR induces mitochondrial dysfunction, generating excessive ROS, downregulates HIF-1α and Higd2a, and reduces VEGFα expression. This exacerbates tumor hypoxia and triggering hypoxic stress, which further activates the DNA damage response, upregulates p53, and induces p21 and Cyclin D1 expression, ultimately causing G1 phase cell cycle arrest

In addition, a notable discovery is the decrease in VEGFα expression and vascular density within tumors following FIR treatment. This finding aligns with the results of *Song et al.*, who reported that temperatures exceeding 42 °C cause damage to the tumor vascular system, reduce blood flow, induce nutrient deprivation, and create a hypoxic environment [52–54]. Meanwhile, a study that irradiated human umbilical vein endothelial cells (HUVECs) with a dose of 0.13 mW/cm^2^ of FIR for 30 min inhibited the proliferation of HUVECs and VEGF-induced extracellular signal-regulated kinase phosphorylation [55]. HIF-1α is a critical regulatory factor that enables cells to adapt to hypoxic environments and serves as a key cellular target of endogenous ROS. It regulates the expression of stress response genes involved in inflammation, metabolism, oxygen delivery, and cell survival [56, 57]. Under normoxic conditions, HIF-1α is rapidly degraded through the pVHL-mediated ubiquitin-proteasome pathway, while hypoxia inhibits this degradation, leading to its accumulation [58, 59]. Hypoxia stabilizes HIF-1α, allowing it to facilitate cellular adaptation to low oxygen levels and promote tumor growth and progression [60]. However, as apoptosis progresses, metabolic and signaling pathways in tumor cells may be altered, resulting in decreased HIF-1α expression [61]. This reduction further weakens its regulatory control over angiogenesis factors such as VEGFα [62, 63], leading to diminished VEGFα levels and exacerbating hypoxia in tumor tissues. Collectively, these changes promote tumor cell apoptosis and suppress tumor growth and progression [64, 65]. Higd2a, identified as a hypoxia sensor and a member of the hypoxia-inducible gene family, typically elevates in expression during the early hypoxia stages to enhance cell survival and decrease hypoxia-induced apoptosis [27, 66]. Furthermore, its promoter region contains a transcription factor binding site (E2F-1) linked to cell cycle regulation [66], which may to cell cycle arrest under severe hypoxia conditions. As a result, hypoxia and oxidative stress are closely associated with apoptosis, with the potential mechanisms of FIR’s effects outlined in Fig. 7. Therefore, we hypothesize that FIR may exhibit greater efficacy against superficial or extremity tumors.

## Conclusion

This study provides a comprehensive understanding of the inhibitory effects of FIR on melanoma cells. Our findings demonstrate that FIR exerts anticancer effects primarily by inducing apoptosis, cell cycle arrest, and hypoxic stress in both in vitro and in vivo models. These results highlight the potential of FIR as a non-invasive melanoma treatment, offering advantages over traditional therapies, including low toxicity, high safety, and strong efficacy. This study provides new insights into the development of tumor treatment strategies that leverage the unique benefits of far-infrared therapy.

## Electronic supplementary material

Below is the link to the electronic supplementary material.

Supplementary Material 1

Supplementary Material 2

## Data Availability

The original data presented in the study are openly available in Genome Sequence Archive (GSA: CRA017581) at https://ngdc.cncb.ac.cn/gsa.
