# Supplementary material for: Induction of apoptosis and hypoxic stress in malignant melanoma cells via graphene-mediated far-infrared radiation
Source: BMC Cancer. 2025 Apr 7;25:620. doi: 10.1186/s12885-025-14031-0 (PMC11974076; doi:10.1186/s12885-025-14031-0)
Supplement: Supplementary file 1 — Supplementary Material 1 [file 12885_2025_14031_MOESM1_ESM.pdf]

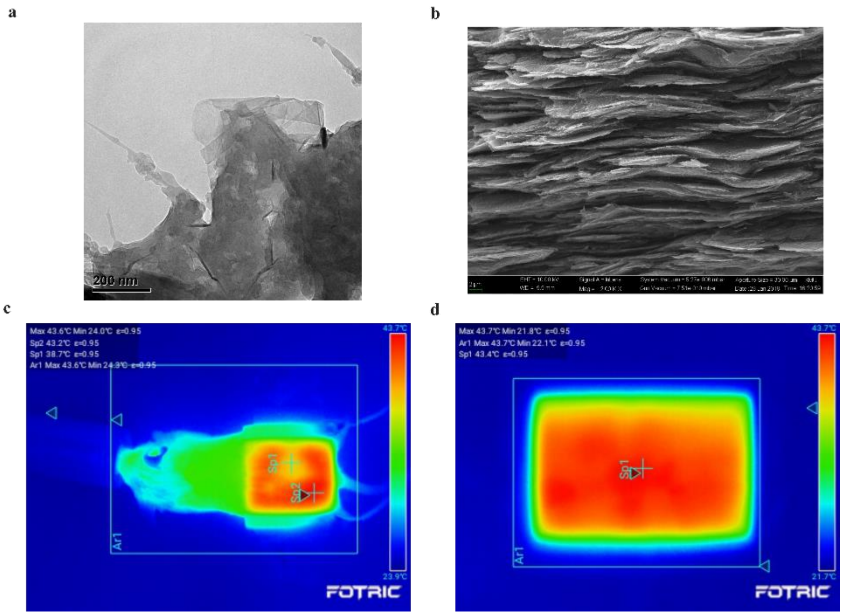

Data1. Characteristics of far-infrared emitting device. a-b) TEM and SEM images of materials from graphene ink. **c)** Schematic diagram of the FIR device and its therapeutic illustration in mice. **d)** Thermal imaging of a far-infrared emitting devices under a power of 5 W.

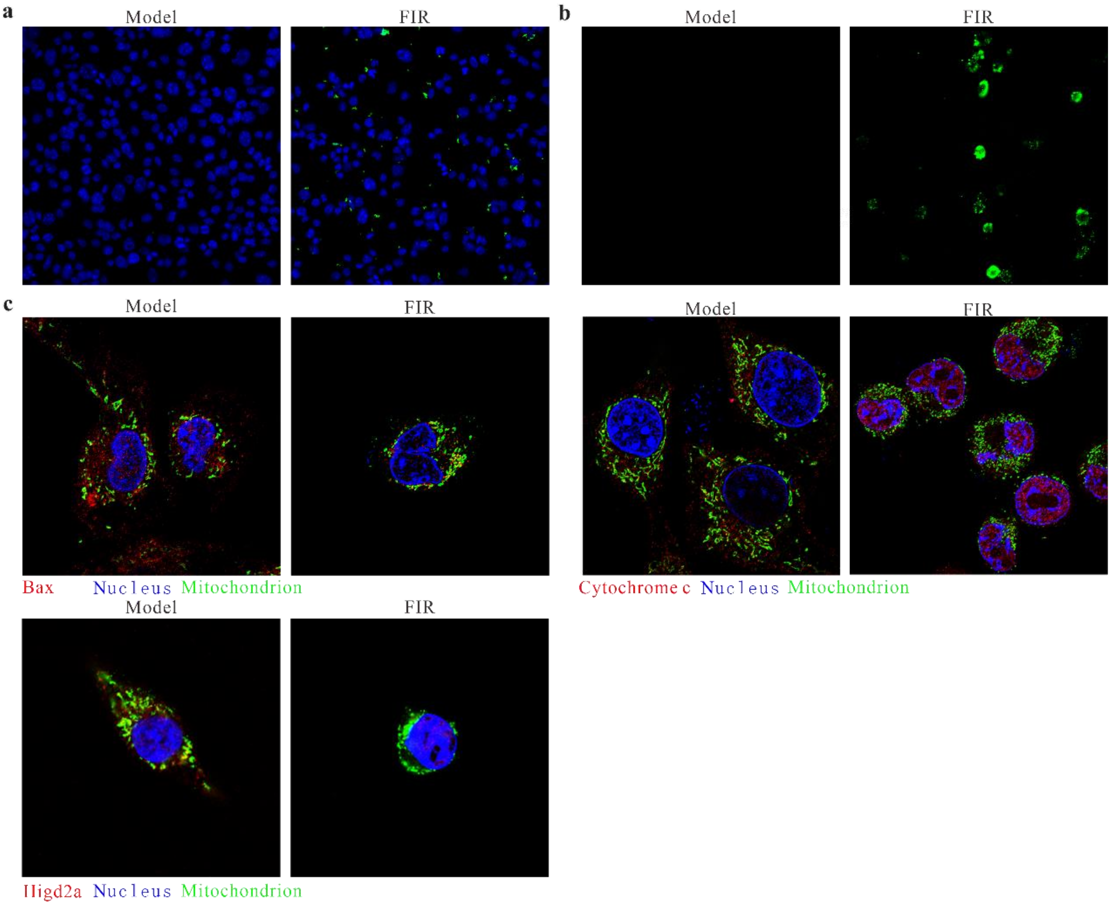

Data2. Microscopic scanning image. a) Images form the TUNEL staining kit to detect apoptotic cell with positive staining in B16F10 cells. **b)** Content of ROS detected by immunofluorescence staining and flow cytometry. **c)** Images of immunofluorescence staining to observe the location changes of Bax, cytochrome c and Higd2a in B16F10 cells.

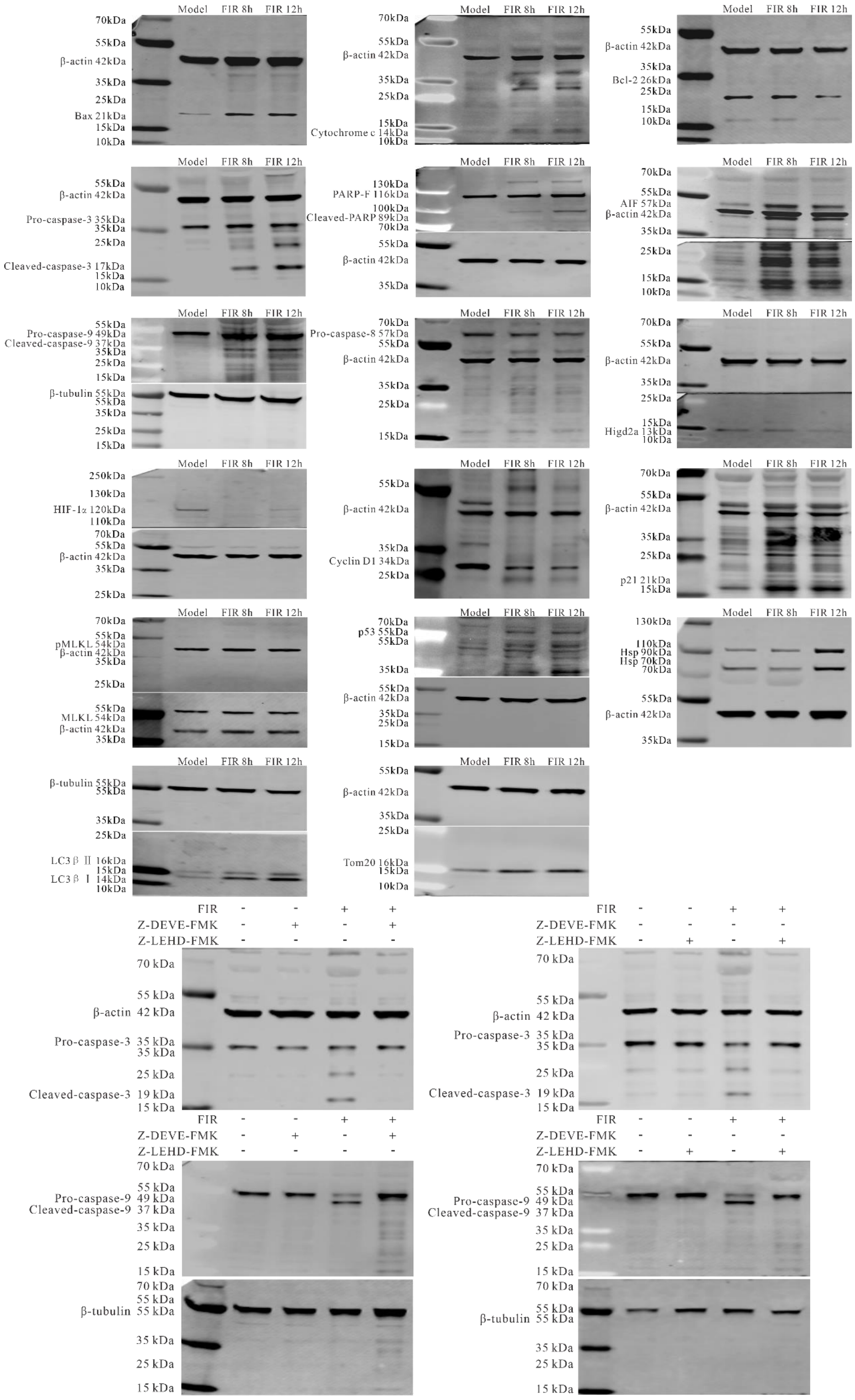

Model FIR 8h FIR 12h
70kDa
55kDa
β-actin 42kDa
35kDa
25kDa
Bax 21kDa
15kDa
10kDa
Model FIR 8h FIR 12h
70kDa
55kDa
β-actin 42kDa
35kDa
25kDa
15kDa
Cytochrome c 14kDa
10kDa
Model FIR 8h FIR 12h
55kDa
β-actin 42kDa
35kDa
Bcl-2 26kDa
25kDa
15kDa
10kDa
Model FIR 8h FIR 12h
55kDa
β-actin 42kDa
Pro-caspase-3 35kDa
35kDa
25kDa
Cleaved-caspase-3 17kDa
15kDa
10kDa
Model FIR 8h FIR 12h
130kDa
PARP-F 116kDa
100kDa
Cleaved-PARP 89kDa
70kDa
55kDa
β-actin 42kDa
35kDa
Model FIR 8h FIR 12h
70kDa
55kDa
AIF 57kDa
β-actin 42kDa
35kDa
25kDa
15kDa
10kDa
Model FIR 8h FIR 12h
55kDa
Pro-caspase-9 49kDa
Cleaved-caspase-9 37kDa
35kDa
25kDa
15kDa
β-tubulin 55kDa
55kDa
35kDa
25kDa
15kDa
Model FIR 8h FIR 12h
70kDa
Pro-caspase-8 57kDa
55kDa
β-actin 42kDa
35kDa
25kDa
15kDa
Model FIR 8h FIR 12h
70kDa
55kDa
β-actin 42kDa
35kDa
25kDa
15kDa
Higd2a 13kDa
10kDa
Model FIR 8h FIR 12h
250kDa
130kDa
HIF-1α 120kDa
110kDa
70kDa
55kDa
β-actin 42kDa
35kDa
25kDa
Model FIR 8h FIR 12h
55kDa
β-actin 42kDa
35kDa
Cyclin D1 34kDa
25kDa
Model FIR 8h FIR 12h
70kDa
55kDa
β-actin 42kDa
35kDa
25kDa
p21 21kDa
15kDa
Model FIR 8h FIR 12h
70kDa
55kDa
pMLKL 54kDa
β-actin 42kDa
35kDa
25kDa
55kDa
MLKL 54kDa
β-actin 42kDa
35kDa
Model FIR 8h FIR 12h
70kDa
p53 55kDa
55kDa
35kDa
55kDa
β-actin 42kDa
35kDa
25kDa
15kDa
Model FIR 8h FIR 12h
130kDa
110kDa
Hsp 90kDa
Hsp 70kDa
70kDa
55kDa
β-actin 42kDa
35kDa
Model FIR 8h FIR 12h
β-tubulin 55kDa
55kDa
35kDa
25kDa
LC3β Ⅱ 16kDa
15kDa
LC3β Ⅰ 14kDa
10kDa
Model FIR 8h FIR 12h
55kDa
β-actin 42kDa
35kDa
25kDa
Tom20 16kDa
15kDa
10kDa
FIR - - + +
Z-DEVE-FMK - + - +
Z-LEHD-FMK - - - -
70 kDa
55 kDa
β-actin 42 kDa
Pro-caspase-3 35 kDa
35 kDa
25 kDa
Cleaved-caspase-3 19 kDa
15 kDa
FIR - - + +
Z-DEVE-FMK - - - -
Z-LEHD-FMK - + - +
70 kDa
55 kDa
β-actin 42 kDa
Pro-caspase-3 35 kDa
35 kDa
25 kDa
Cleaved-caspase-3 19 kDa
15 kDa
FIR - - + +
Z-DEVE-FMK - + - +
Z-LEHD-FMK - - - -
70 kDa
55 kDa
Pro-caspase-9 49 kDa
Cleaved-caspase-9 37 kDa
35 kDa
25 kDa
15 kDa
70 kDa
55 kDa
β-tubulin 55 kDa
35 kDa
25 kDa
15 kDa
FIR - - + +
Z-DEVE-FMK - - - -
Z-LEHD-FMK - + - +
70 kDa
55 kDa
Pro-caspase-9 49 kDa
Cleaved-caspase-9 37 kDa
35 kDa
25 kDa
15 kDa
70 kDa
55 kDa
β-tubulin 55 kDa
35 kDa
25 kDa
15 kDa

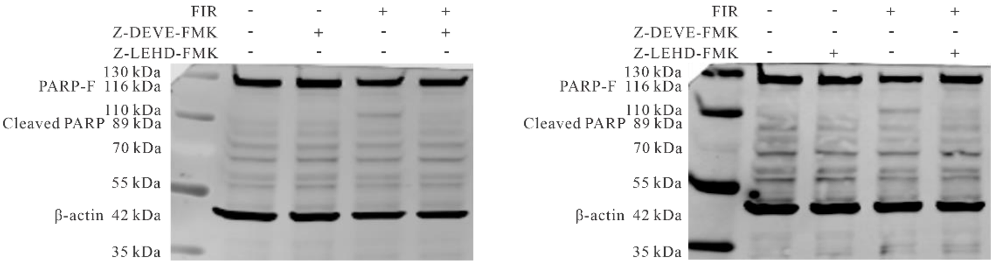

Data3. Western blot analysis at 8 h and 12 h after treatment to detect protein expression.

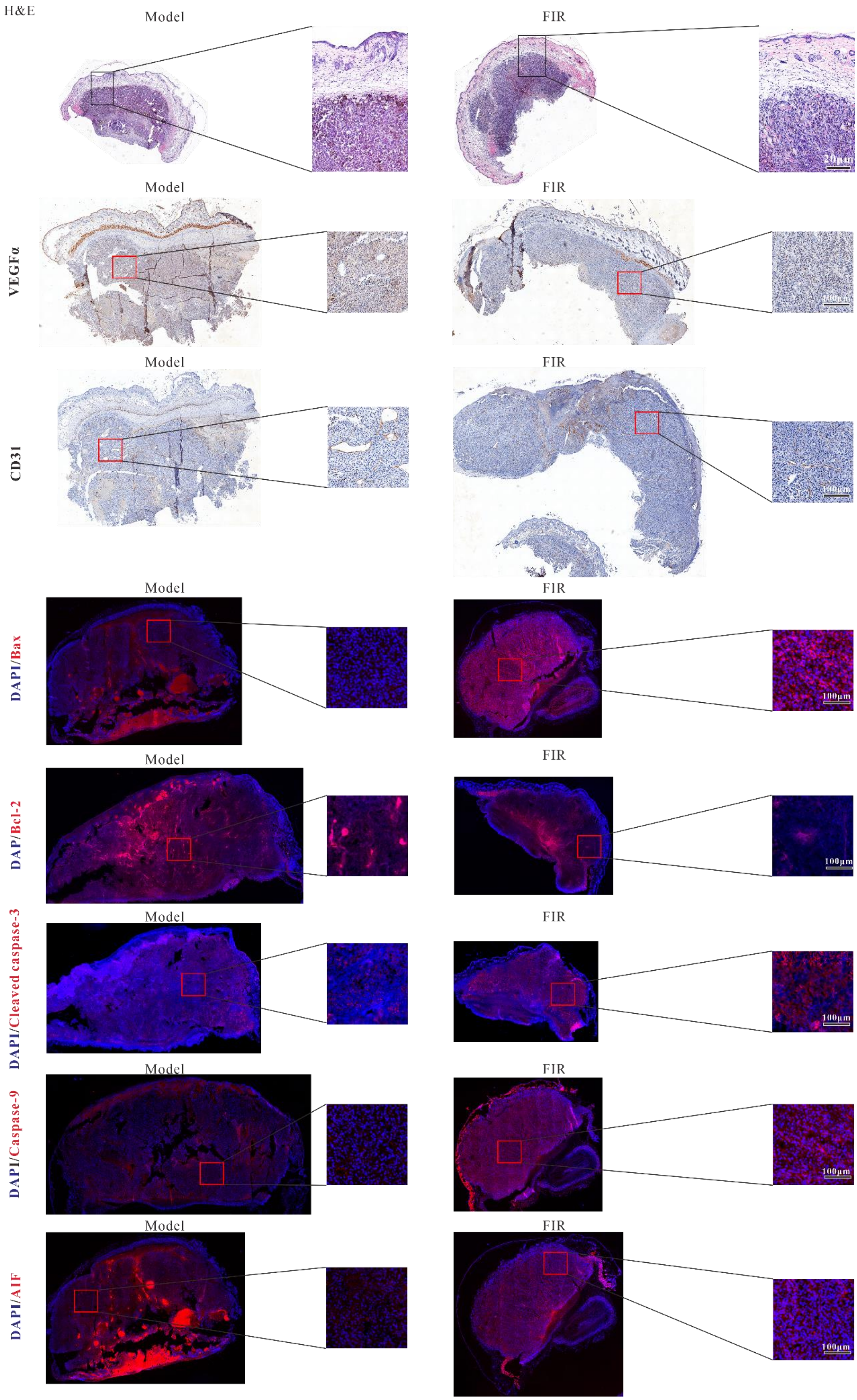

H&E
Model
FIR
20μm
VEGFα
Model
FIR
CD31
Model
FIR
100μm
DAPI/Bax
Model
FIR
100μm
DAP/Bcl-2
Model
FIR
100μm
DAPI/Cleaved caspase-3
Model
FIR
100μm
DAPI/Caspase-9
Model
FIR
100μm
DAPI/AIF
Model
FIR
100μm

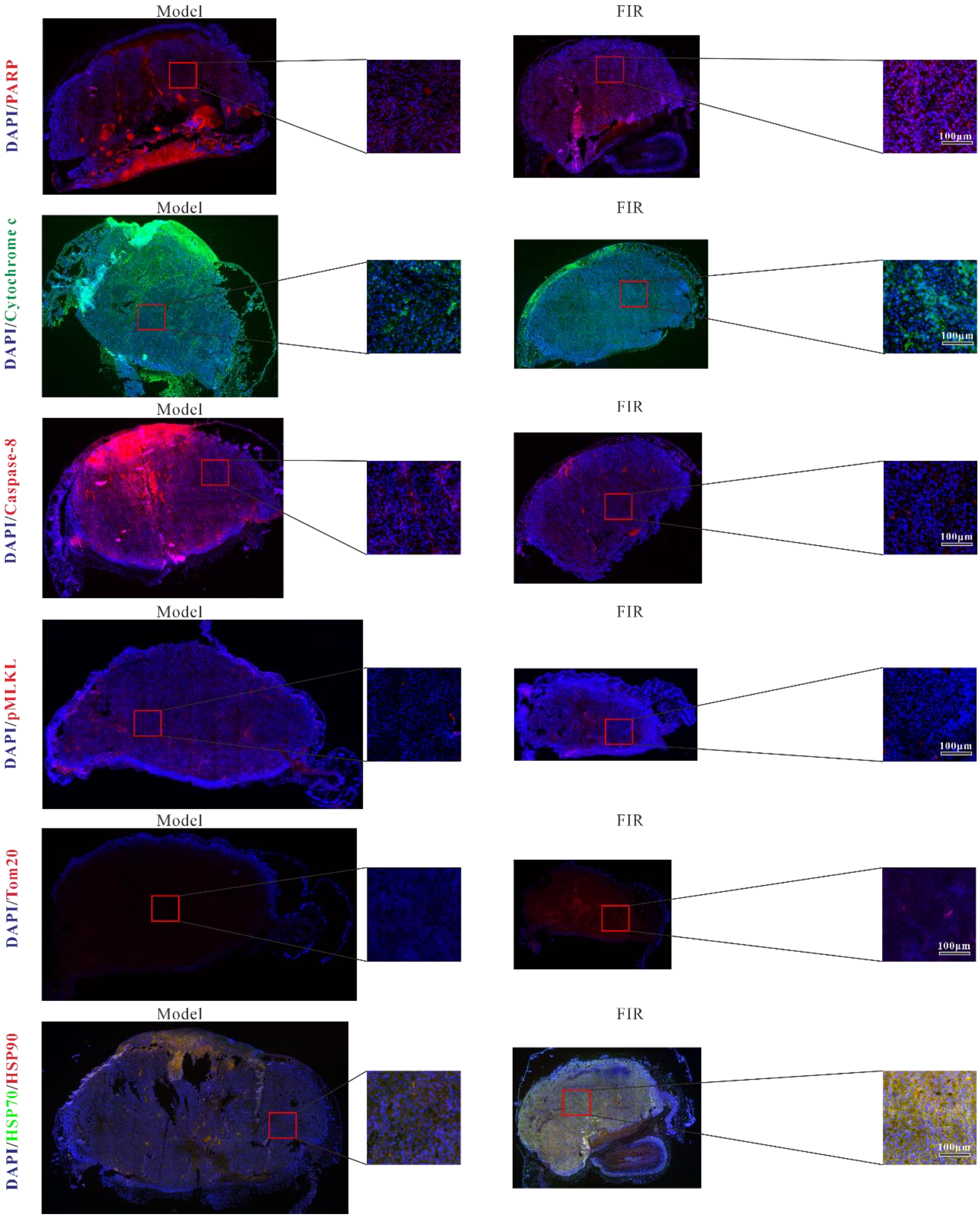

Data4. Performing hematoxylin and eosin (H&E) staining and immunohistofluorescence staining on the tumor tissues.

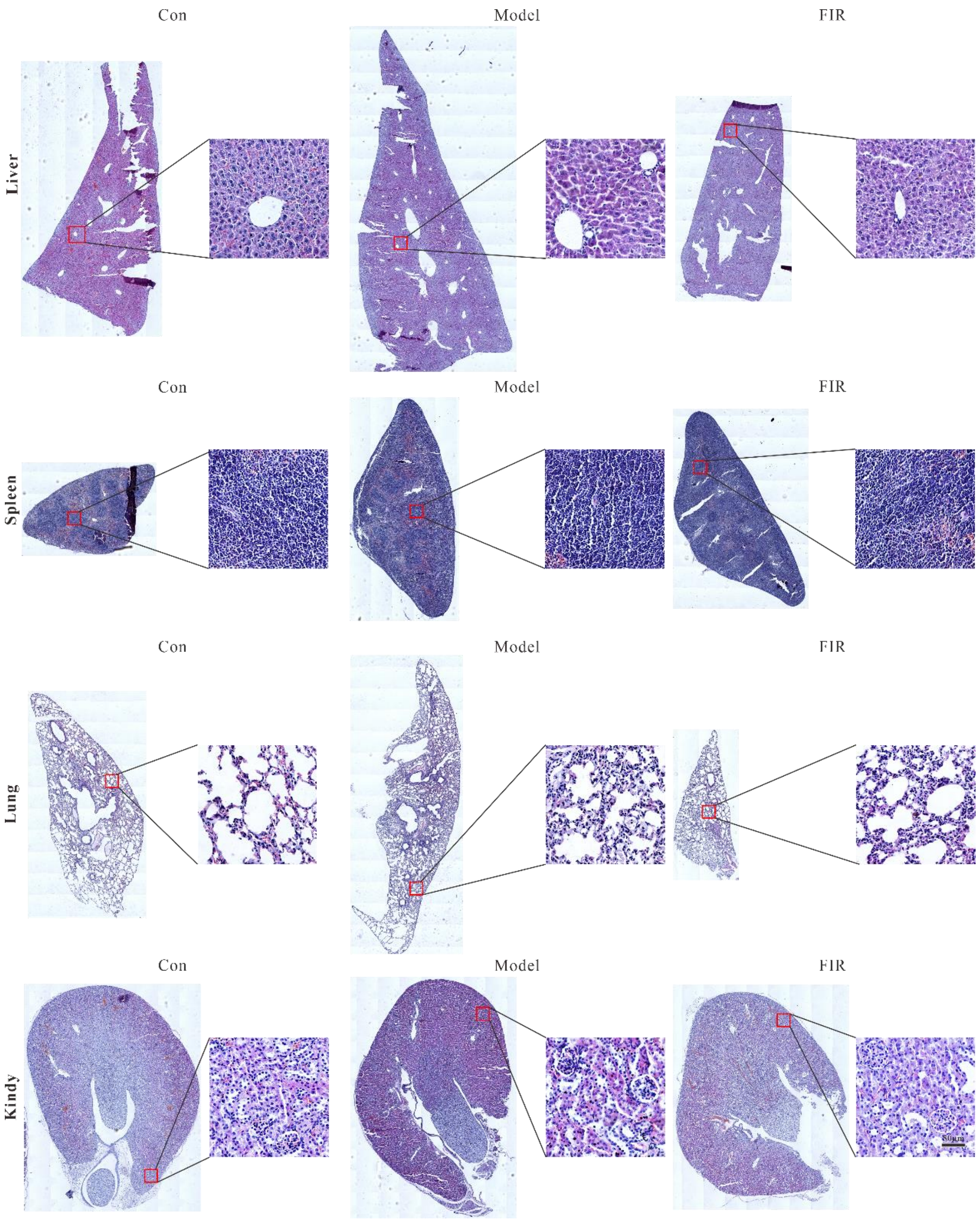

Data5. Histopathological features of vital organs (spleen, lung, liver, and kidney) in the Control, Model and FIR groups.
