# Supplementary material for: Induction of apoptosis and hypoxic stress in malignant melanoma cells via graphene-mediated far-infrared radiation
Source: BMC Cancer. 2025 Apr 7;25:620. doi: 10.1186/s12885-025-14031-0 (PMC11974076; doi:10.1186/s12885-025-14031-0)
Supplement: Supplementary file 2 — Supplementary Material 2 [file 12885_2025_14031_MOESM2_ESM.docx]

Induction of Apoptosis and Hypoxic Stress in Malignant Melanoma Cells via Graphene-Mediated Far-Infrared Radiation

1 Department of Dermatology, Xiang'an Hospital of Xiamen University, School of Medicine, Xiamen University, Xiamen 361101, China.

^2^ New Cornerstone Science Laboratory, State Key Laboratory for Physical Chemistry of Solid Surfaces, Collaborative Innovation Center of Chemistry for Energy Materials, and National & Local Joint Engineering Research Center of Preparation Technology of Nanomaterials, College of Chemistry and Chemical Engineering, Xiamen University, Xiamen 361005, China

^3^ Innovation Laboratory for Sciences and Technologies of Energy Materials of Fujian Province (IKKEM), Xiamen 361101, China

4 Department of cardiology, Shaoxing Central Hospital, Shaoxing, 312030, China.

5 Jinhua Fifth Hospital, College of Mathematical Medicine, Zhejiang Normal University, Jinhua 321004, China.

*Corresponding authors. E-mails: shanshijun2023@163.com; linachensx@126.com;

binghuiwu@xmu.edu.cn

Contributing authors:1950927920@qq.com; [1007090899@qq.com](mailto:1007090899@qq.com); [fuwenxing@xmu.edu](mailto:fuwenxing@xmu.edu); [409352724@qq.com](mailto:409352724@qq.com); [867216897@qq.com](mailto:867216897@qq.com); [xty9829@163.com](mailto:xty9829@163.com)[; binghuiwu@xmu.edu.cn;](mailto:;%20binghuiwu@xmu.edu.cn;%20nfzheng@xmu.edu.cn)  linachensx@126.com; [shanshijun2023@163.com](mailto:shanshijun2023@163.com)


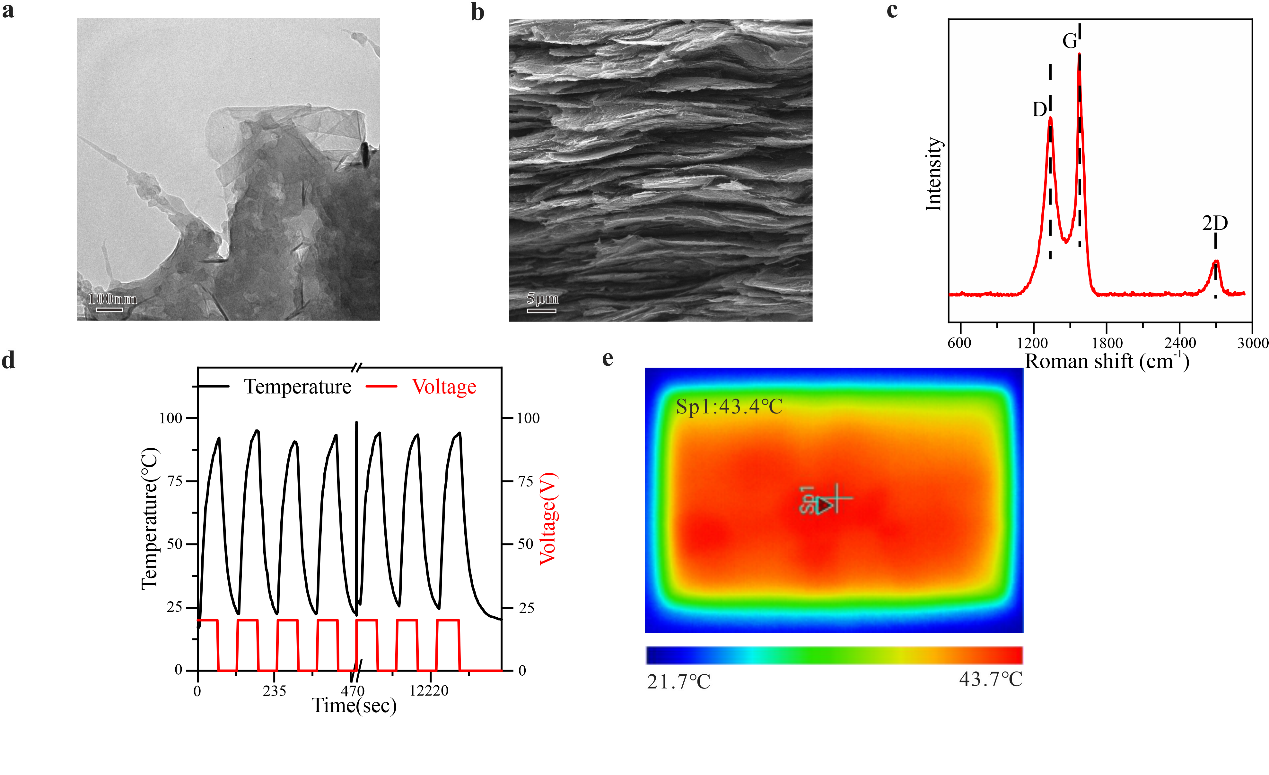


**Fig S1. Characteristics of far-infrared emitting devices. a-b)** TEM and SEM images of materials derived from graphene ink. **c)** Raman characterization of graphene films. The D peak originates from carbon black nanoparticles, while the G and 2D peaks are attributed to single-layer graphene and multilayers with fewer than 10 layers, respectively. **d)** Temperature curve of graphene films subjected to a square wave voltage ranging from 0 to 20 V. **e)** Thermal imaging of a far-infrared emitting device under a power of 5 W. Data are presented as the mean ± SD of three independent experiments, with significance levels denoted as *P < 0.05, **P < 0.01, ***P < 0.001, and ****P < 0.0001.


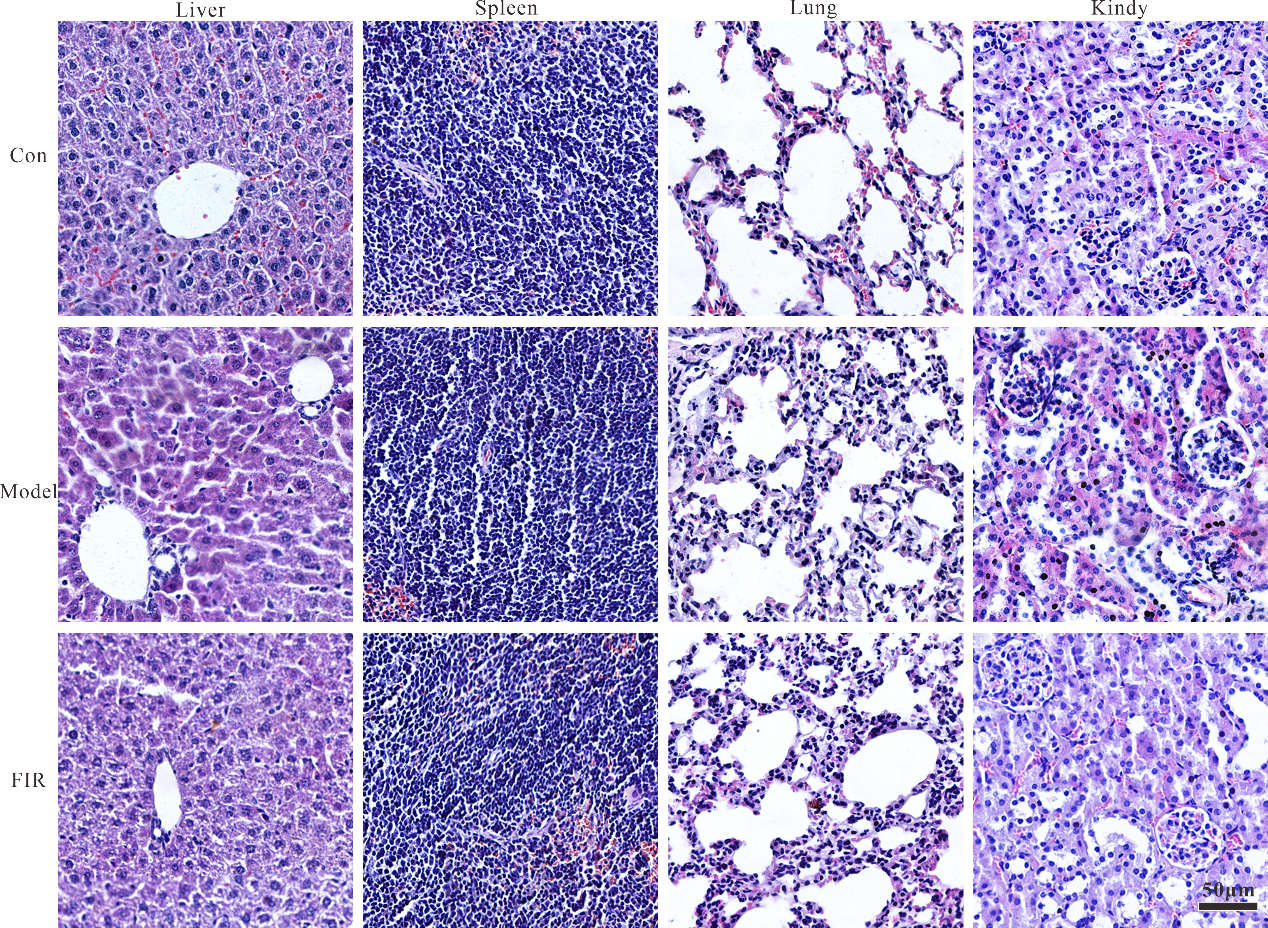
 **Fig S2.**  Histopathologic features of vital organs (spleen, lung, liver, and kidney) in the Control, Model, and FIR groups. Data are presented as the mean ± SD of three independent experiments, with significance levels denoted as *P < 0.05, **P < 0.01, ***P < 0.001, and ****P < 0.0001.


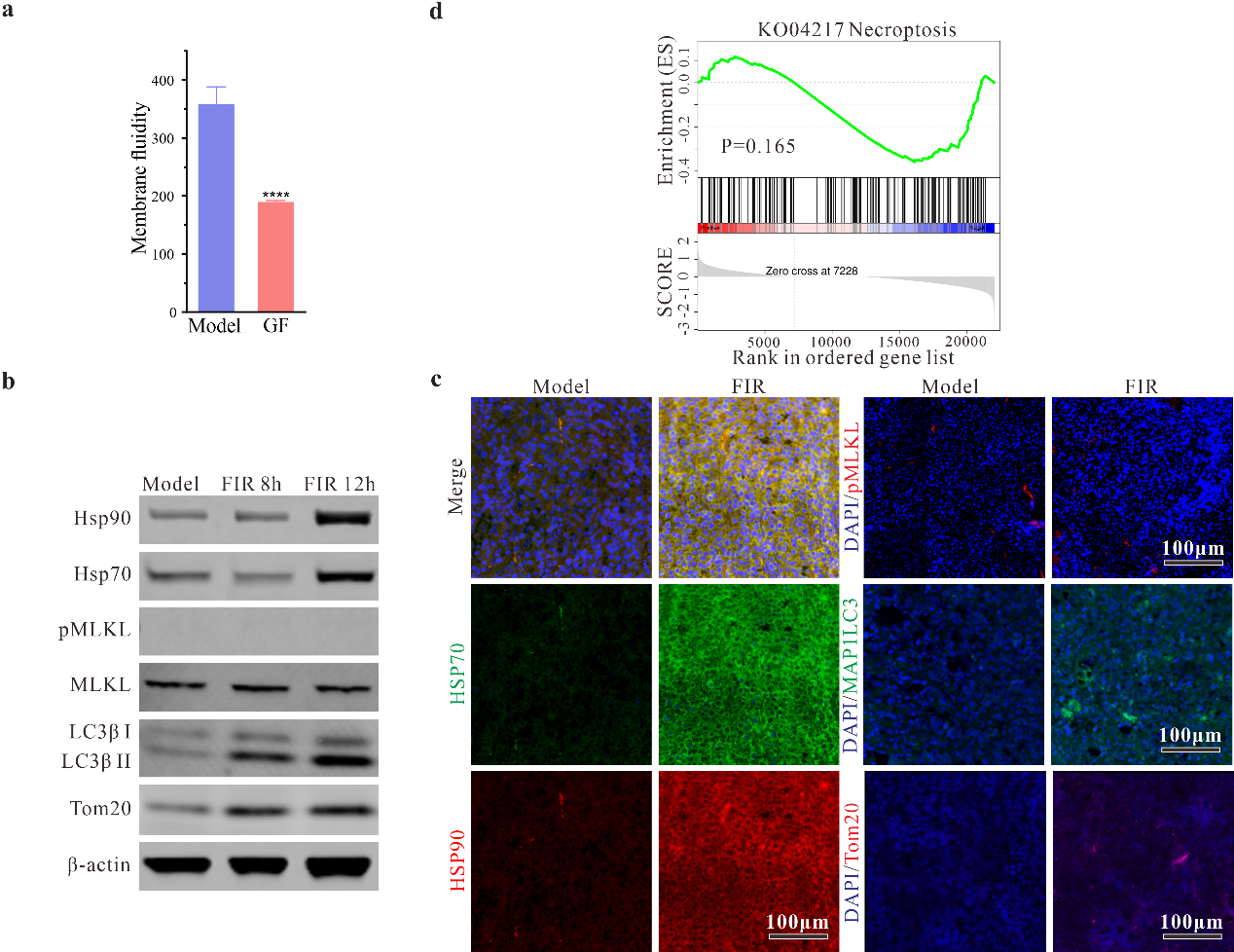


**Fig S3. The impact of FIR treatment on the regulation of immune response, necrosis, and autophagy functions in melanoma cells. a)** Assessment of membrane fluidity in B16F10 cells subjected to FIR treatment. **b)** Protein expression in B16F10 cells determined by Western blotting. **c)**  Immunohistochemical fluorescence detection of HSP70, HSP90, MAP1LC3, and Tom20 expression levels. **d)** GSEA analysis of B16F10 cells treated with FIR compared to the Model group. Data are presented as the mean ± SD of three independent experiments, with significance levels denoted as *P < 0.05, **P < 0.01, ***P < 0.001, and ****P < 0.0001.
